# High level transgenic expression of soybean (*Glycine max*) GmERF and Gmubi gene promoters isolated by a novel promoter analysis pipeline

**DOI:** 10.1186/1471-2229-10-237

**Published:** 2010-11-04

**Authors:** Carlos M Hernandez-Garcia, Robert A Bouchard, Paul J Rushton, Michelle L Jones, Xianfeng Chen, Michael P Timko, John J Finer

**Affiliations:** 1 Department of Horticulture and Crop Science, OARDC/The Ohio State University, 1680 Madison Ave., Wooster, OH 44691 USA; 2 Department of Biology, University of Virginia, Charlottesville, VA 22904 USA; 3 Department of Microbiology, University of Virginia Health Systems, Charlottesville, VA 22908 USA; 4 Department of Biology and Microbiology, South Dakota State University, Brookings, SD 57007 USA; 5 USACE, Environmental Lab, ERDC, 3909 Halls Ferry Road, Vicksburg, MS 39180 USA

## Abstract

**Background:**

Although numerous factors can influence gene expression, promoters are perhaps the most important component of the regulatory control process. Promoter regions are often defined as a region upstream of the transcriptional start. They contain regulatory elements that interact with regulatory proteins to modulate gene expression. Most genes possess their own unique promoter and large numbers of promoters are therefore available for study. Unfortunately, relatively few promoters have been isolated and characterized; particularly from soybean (*Glycine max*).

**Results:**

In this research, a bioinformatics approach was first performed to identify members of the *Gmubi *(*G.max *ubiquitin) and the *GmERF *(*G. max *Ethylene Response Factor) gene families of soybean. Ten Gmubi and ten GmERF promoters from selected genes were cloned upstream of the *gfp *gene and successfully characterized using rapid validation tools developed for both transient and stable expression. Quantification of promoter strength using transient expression in lima bean (*Phaseolus lunatus*) cotyledonary tissue and stable expression in soybean hairy roots showed that the intensity of *gfp *gene expression was mostly conserved across the two expression systems. Seven of the ten Gmubi promoters yielded from 2- to 7-fold higher expression than a standard CaMV35S promoter while four of the ten GmERF promoters showed from 1.5- to 2.2-times higher GFP levels compared to the CaMV35S promoter. Quantification of GFP expression in stably-transformed hairy roots of soybean was variable among roots derived from different transformation events but consistent among secondary roots, derived from the same primary transformation events. Molecular analysis of hairy root events revealed a direct relationship between copy number and expression intensity; higher copy number events displayed higher GFP expression.

**Conclusion:**

In this study, we present expression intensity data on 20 novel soybean promoters from two different gene families, *ubiquitin *and *ERF*. We also demonstrate the utility of lima bean cotyledons and soybean hairy roots for rapid promoter analyses and provide novel insights towards the utilization of these expression systems. The soybean promoters characterized here will be useful for production of transgenic soybean plants for both basic research and commercial plant improvement.

## Background

With the increasing amount of biological information derived from genome sequencing projects of several plant species [1,2], opportunities exist for functional analysis of those sequences using a combination of computational approaches and various methods of wet laboratory analyses of gene expression. The recent release of the soybean genome [3] has tremendously facilitated computational genome-wide analyses of the soybean genome and identification of specific DNA sequences, which need to be validated using functional analysis tools. The availability of the soybean genome has also provided unprecedented access to sequences for a wide range of promoters from diverse gene families, which will lead to a better understanding of the regulation of gene expression and the discovery of novel soybean promoters for use in basic research and applied crop biotechnology.

Promoters are the primary regulators of gene expression at the transcriptional level and are key to controlling transgenes in transgenic organisms [4]. The use of one or only a few different promoters to direct expression of different genes in transgene stacks can lead to homology-based gene silencing and unpredictable transgene expression in transgenic plants [5]. Consequently, it is absolutely necessary to increase the availability of different promoters for plant transformation. Although the constitutive highly-expressed Cauliflower Mosaic Virus 35S (CaMV35S) promoter is commonly used for gene regulation in plants, different plant genomes can provide additional useful native plant promoters ranging from highly-expressing constitutive to tissue-specific and inducible. Likewise, analyses of native promoters will most likely reveal a large variety of heretofore undiscovered *cis*-regulatory elements, which will increase our understanding of gene expression regulation [6]. Although several plant promoters are available as an alternative to the CaMV35S promoter, very few soybean promoters have been isolated and extensively characterized in soybean [7-9], in spite of the world-wide economic impact of this crop.

We recently reported the isolation and characterization of a *Glycine max *polyubiquitin (Gmubi) promoter, which leads to high constitutive levels of both transient [10] and stable gene expression in various tissues of transgenic soybeans [7]. Other plant ubiquitin promoters have also been isolated and characterized in a wide variety of plant species [4]. Particularly, ubiquitin promoters from rice [11] and maize [12] have been extensively characterized and frequently used in both basic research and in the production of commercial transgenics. Ubiquitin promoters typically drive strong constitutive gene expression, which is especially high in young tissues, vascular tissues and pollen [13]. The enhancement of gene expression from the presence of the leading intron in the different ubiquitin promoters has also received considerable attention [14]. In spite of the emphasis on the use of ubiquitin promoters, most studies to date have relied on single promoter sequences isolated from different plant species [15-17]. However, the ubiquitin gene family is quite large in most plants and isolation and characterization of different ubiquitin promoters, from the same plant, could serve as a source of additional promoters and provide useful information on how different *ubiquitin *genes are differentially regulated.

As ubiquitin promoters tend to drive constitutive gene expression, additional promoter sequences from inducible genes may also be of interest [18]. The Ethylene Response Factor (ERF) gene family encodes a large group of transcription factors characterized by the presence of a single AP2/ERF domain [19]. ERF proteins play important roles in ethylene-mediated gene transcription [20] and in a wide range of biotic and abiotic stress responses such as pathogen attack [21], drought tolerance, salt tolerance and low temperatures [22,23]. The *ERF *genes therefore could be excellent sources for inducible promoters, which most likely contain interesting *cis*-regulatory elements within their sequences.

Promoter characterization typically involves the introduction and analysis of DNA constructs containing promoters fused to a reporter gene. Temporal and tissue-specific expression of the reporter gene can then be directly observed and quantified in transgenic plant tissues. Although soybean transformation was first reported many years ago [24-26], it remains consistent but inefficient [27] and it may not be entirely suitable for medium- to high-throughput analysis of soybean promoters. Due to this limitation, analyses of soybean promoters and their *cis*-regulatory elements are often performed using heterologous plant expression systems such as *Arabidopsis *and tobacco [9,28,29]. Analyses using heterologous systems have value but validation of soybean promoters in soybean [7,8], or at least in another member of the *Fabaceae *family, is preferred as heterologous systems may not accurately reflect promoter strength and specificity [30-32].

For rapid analysis of promoters, transient gene expression offers many advantages and some disadvantages compared with the use of stably-transformed tissues. Transient expression can be detected as early as 2 h post DNA introduction in soybean tissues [33], which is quite useful for rapid estimation of promoter activity. Depending on the method for DNA introduction [34], different tissue types can be targeted for gene delivery, allowing increased flexibility in construct evaluations. In our laboratory, transient expression has been successfully used for evaluation of soybean promoter variants [10], but these evaluations were performed using lima bean (*Phaseolus lunatus*) cotyledons. Transient expression analysis for promoter validation using soybean cotyledons as an alternate target to lima bean cotyledons has not been previously reported.

For evaluation of constructs in stably-transformed soybean tissues, the production of hairy roots provides the most rapid and efficient method for generation of transgenic soybean tissues. Soybean hairy root cultures induced by *Agrobacterium rhizogenes *have been successfully used for rapid analysis of soybean cyst nematode infestation [35], improvement of genetic transformation efficiencies [36] and analysis of phenolic metabolism [28,37]. As an alternate approach, composite plants [38] consisting of hairy roots on non-transgenic shoots are also useful for rapid evaluation of gene expression in stably transformed soybean tissues [39]. Previous molecular analyses conducted on soybean hairy roots have revealed the presence of high copy number integrations [35,36,38], although the relationship between high copy number insertions and gene expression in hairy roots has not been reported.

With the aim of discovering unique and useful soybean promoters with potential applications in both basic research and crop improvement, we here identify, clone and validate 20 novel soybean promoters from the *ubiquitin *and *ERF *gene families. We present two different and complementary promoter validation tools based on transient expression in lima bean cotyledons and production of stably-transformed soybean hairy roots. Quantitative gene expression analysis of these 20 new soybean promoters using 2 different promoter validation tools allows us to greatly expand the toolbox of available soybean promoters.

## Results

### Phylogenetic analysis of the *Gmubi *and *GmERF *genes

**Figure 1:**
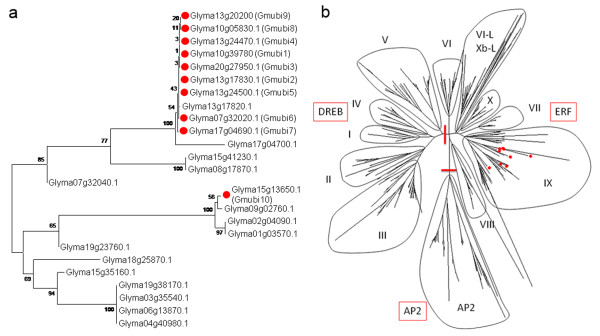
**The *ubiquitin *and *ERF *gene families in soybean**. (a) Phylogenetic tree of soybean *ubiquitin *genes constructed using the amino acid sequences of 25 genes containing at least one ubiquitin-coding unit (Additional file 1. (b) Phylogenetic tree of soybean *ERF *genes constructed using the predicted amino acid sequences of the AP2/ERF domain of 359 ERF proteins (Additional file 2). Eleven ERF subgroups and the AP2 family genes are shown. Red lines divide the three major subfamilies. The amino acid sequences were aligned by Clustal W and the phylogenetic trees were constructed using MEGA 4.0 and the Neighbor-Joining method. The genes used for promoter isolation are shown with red circles.

Phylogenetic analyses of the gene models in the soybean genome revealed at least 46 genes whose predicted amino acid sequences contained at least one ubiquitin-coding unit (Additional file 1). Of these, 25 genes were similar to the ubiquitin gene family that includes Gmubi1-10 (Figure 1a). The other 21 genes contained a lower number of ubiquitin-like coding units more similar to those found in other proteins such as the apoptotic regulator Scythe and the adaptor molecule RAD23 [40,41]. The *Gmubi1-9 *genes all belong to the same polyubiquitin-containing family; whereas, the *Gmubi10 *gene belongs to a different, small monoubiquitin-containing subfamily (Figure 1a).

Phylogenetic analyses of the ERF/AP2 genes from soybean revealed a total of 371 genes, which could be annotated as AP2/ERF genes (Additional file 2). Of these, 12 genes were not incorporated into the phylogenetic tree as they were either too divergent or incorrectly predicted (Glyma01g22260.1, Glyma02g11060.1, Glyma05g07840.1, Glyma08g24110.1, Glyma11g05450.1, Glyma14g00600.1, Glyma15g25120.1, Glyma17g17010.1, Glyma18g01030.1, Glyma19g43260.1, Glyma19g43260.2, Glyma19g45390.1). A total of 359 ERF/AP2 genes were retained, including the ten chosen for this study. The soybean AP2/ERF family is broadly similar to that from other higher plant species and can be subdivided into the ERF and AP2 subfamilies (Figure 1b). Similar to *Arabidopsis *[19] and tobacco [42], the ERF family could be further subdivided into the DREB (groups I-V) and the ERF subfamilies (groups VI-X). One additional subfamily was apparent and may be related to the members of group VI-L and Xb-L as these proteins were omitted from both the *Arabidopsis *and tobacco analyses. This phylogenetic analysis provides a framework for the study of promoters from other members of the soybean AP2/ERF multigene family and illustrates the phylogenetic positions of the 10 group IX *GmERF *genes used for promoter isolation in this study. The *GmERF1-10 *genes were chosen as they are likely to be wound- and/or jasmonate-inducible based on their phylogenetic position [42].

### Evaluation of soybean and lima bean cotyledons for transient expression analysis

**Figure 2:**
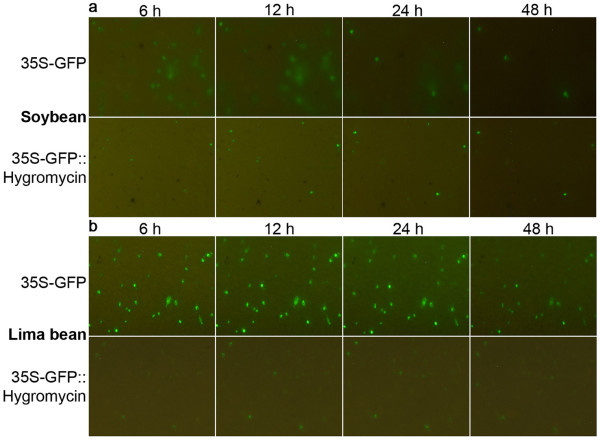
**Transient GFP expression using soybean and lima bean cotyledons**. Soybean cotyledons (a) and lima bean cotyledons (b) were transformed with 35S-GFP [10] and 35S-GFP::Hygromycin [43] plasmids. Numbers on top represent hours (h) after bombardment.

**Figure 3:**
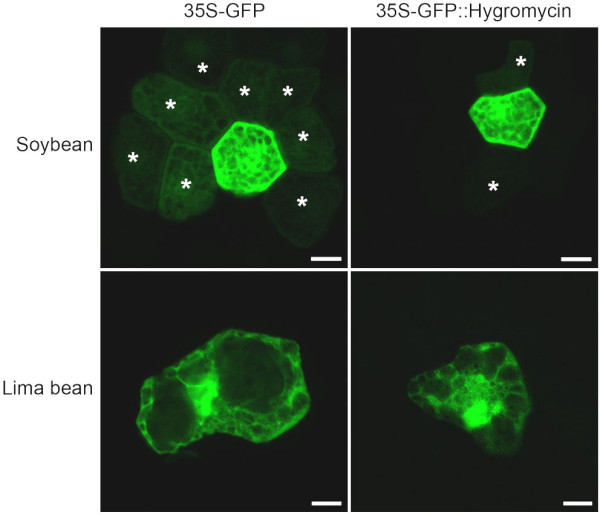
**Confocal microscopy of GFP expression in bombarded soybean and lima bean cotyledons**. The analysis was performed 10 h after DNA introduction. The 35S-GFP and 35S-GFP::Hygromycin plasmids were previously described by Chiera et al [10] and Chiera et al [43], respectively. Asterisks mark GFP-expressing cells adjacent to single transformed cells. Bars are equivalent to 10 μm.

Transient gene expression in soybean and lima bean cotyledons was conducted and compared with the initial aim of developing a soybean-based transient expression system for validation of soybean promoters. Introduction of the 35S-GFP construct into soybean cotyledons resulted in relatively high levels of gene expression, followed by a very rapid decline (Figure 2). In soybean, the GFP appeared to diffuse from the targeted cells into the surrounding cells a few hours after bombardment. GFP was minimally detected using our automated image collection system ~48 h after bombardment (Figure 2a). Time-lapse animations of GFP expression tracked for 100 h in bombarded soybean cotyledons clearly showed the rapid loss of GFP in the primary targeted cells and the apparent diffusion to the surrounding cells (Additional file 3). In contrast, in lima bean cotyledonary cells, transient GFP expression modulated by the 35S-GFP construct appeared to remain localized in single cells (Figure 2b) and time-lapse animations revealed that GFP was visible for over 100 h (Additional file 3). Confocal microscopy of soybean cotyledonary cells, conducted 10 h after bombardment with the 35S-GFP plasmid confirmed high levels of GFP in the cytoplasm and nuclei of the main targeted cells, but also low levels of GFP in the cytoplasm and nuclei of the adjacent cotyledonary cells (Figure 3). Confocal microscopy also confirmed restriction of GFP to the main targeted cell in bombarded lima bean cotyledonary tissues (Figure 3). This analysis also revealed basic differences in cotyledon cell morphology between lima bean and soybean. For example, soybean cotyledonary cells were considerably smaller and more regularly-shaped than lima bean cells and contained smaller vacuoles (Figure 3).

To investigate if the apparent GFP diffusion visualized in soybean cotyledonary cells was related to the small size of GFP, a translational fusion of GFP::Hygromycin [43] was introduced into both soybean and lima bean cotyledons. Although GFP levels and the numbers of GFP-expressing cells were considerably lower than obtained earlier with the 35S-GFP introduction, GFP expression from the translational fusion remained in the targeted cells longer in both plants and was detected until over 100 h after transformation (Figure 2a-b and data not shown). Confocal microscopy of soybean cotyledons, bombarded with the GFP::Hygromycin translational fusion confirmed strong GFP expression in the cytoplasm and nuclei of targeted cells but a clear reduction of GFP levels in the adjacent cells (Figure 3). Confocal analysis of lima bean cotyledons showed high levels of GFP in the cytoplasm and nuclei of targeted cells and no detectable GFP levels in the adjacent cells (Figure 3).

### Transient expression analysis of promoters using lima bean cotyledons

**Figure 4:**
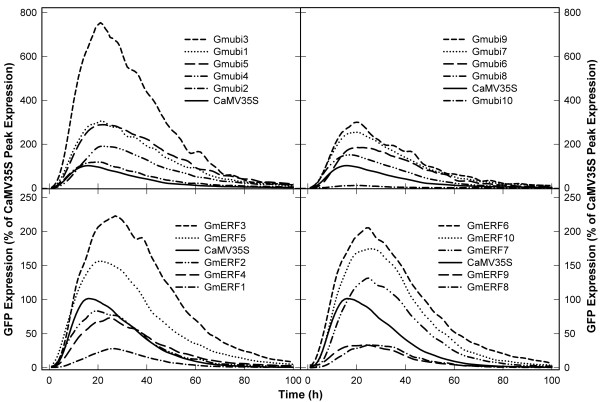
**Profiles of transient GFP expression driven by the Gmubi and GmERF promoters**. Images were collected every hour for 100 h and GFP quantified using ImageJ. GFP expression levels for each promoter are reported as the percent of peak GFP expression obtained with the CaMV35S promoter.

The upstream regions of gene coding sequences for 10 *Gmubi *and 10 *GmERF *genes (Figure 1) were cloned 5' to the *gfp *gene and rapidly characterized. Profiles of transient GFP expression were generated for all 20 soybean promoters, along with the CaMV35S promoter, using lima bean cotyledons as target tissue (Figure 4). GFP expression was first detectable in cotyledonary cells 2-3 h after DNA introduction and became almost undetectable with our automated image collection and analysis system [44] ~100 h after DNA delivery.

The transient expression profiles were mostly similar for all the Gmubi promoters regardless of promoter strength. However, GFP expression peaks for the strong Gmubi promoters appeared to be reached later than the low-expressing promoters or the CaMV35S promoter. Most of the Gmubi promoters gave rise to exceptionally high levels of transient GFP expression based on a comparison to the CaMV35S promoter; the y-axis in Figure 4 is the percent of peak CaMV35S expression. The Gmubi1, Gmubi3, Gmubi4, Gmubi5, Gmubi6, Gmubi7 and Gmubi9 promoters displayed a ~2-7-fold increase in expression over levels obtained with the CaMV35S promoter (Figure 4). The Gmubi2 and Gmubi8 promoters showed similar levels of transient GFP expression compared with the CaMV35S, while use of the Gmubi10 promoter resulted in very low levels of GFP expression.

The transient expression profiles generated for the GmERF promoters also showed a range of promoter strengths but reasonable consistency in the timing of peak expression (Figure 4). The times for peak GFP expression driven by the GmERF promoters were more variable than those observed for the Gmubi promoters but were consistently later than CaMV35S-driven GFP peak (Figure 4). Although many of the GmERF promoters resulted in lower GFP levels than the Gmubi promoters, some gave higher expression than the CaMV35S promoter. The GmERF3, GmERF5, GmERF6, and GmERF10 promoters exhibited ~1.5-2.2-times higher GFP levels compared to the CaMV35S promoter. The GmERF2, GmERF4 and GmERF7 promoters showed similar GFP levels to CaMV35S, while GmERF1, GmERF8 and GmERF9 promoters gave rise to lower levels of transient GFP expression (Figure 4).

### Stable expression analysis using soybean hairy roots

In addition to analysis of promoter activity using transient expression, promoter strength was assessed in stably-transformed soybean hairy roots. One week after *A. rhizogenes*-inoculation of soybean cotyledons, numerous small cell clusters composed of both GFP- and non GFP-expressing cells were evident in the wounded sites located on the abaxial side of the inoculated cotyledons. Approximately two weeks after inoculation, the cotyledons formed numerous roots, which were 72% GFP-positive (Table 1). Although the clear majority of GFP-expressing root events appeared to express GFP homogeneously, formation of chimeric roots was occasionally observed. Most hairy roots were relatively prolific and grew quickly following subculture to fresh OMS medium. Secondary roots could be excised and used to generate additional clonal tissues for analysis.

**Table 1:** Average of primary hairy roots expressing GFP mediated by promoter constructs

Number of roots analyzed	GFP expression	
	
	Positive (+)	Negative (-)
195	140 (72%)	55 (28%)

**Figure 5:**
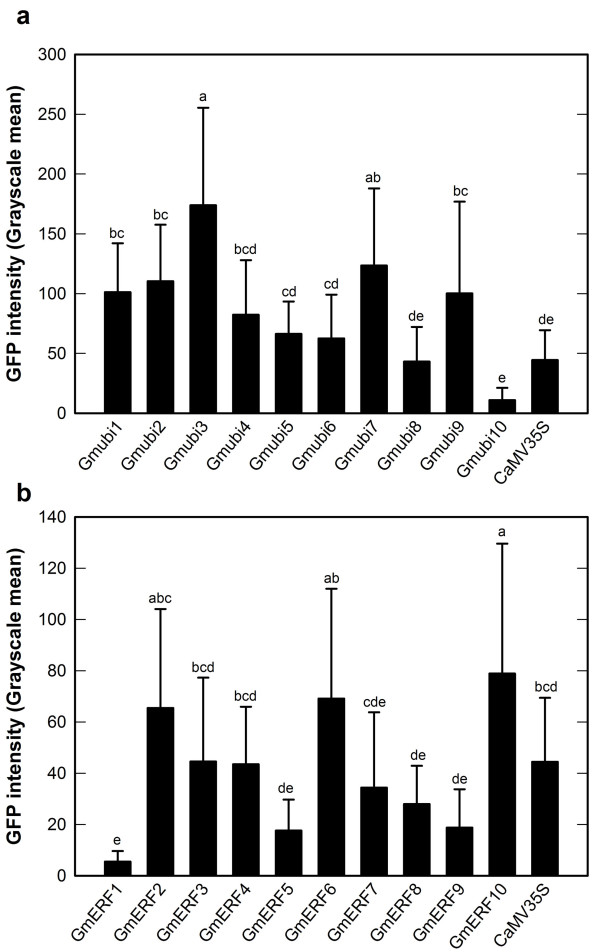
**Analysis of GFP intensity in hairy roots induced with *A. rhizogenes *harboring different promoter constructs**. (a) Gmubi promoters. (b) GmERF promoters. GFP intensity is presented as grayscale values. Values are means ± SD. Letters on top of each column were generated by the Tukey's Studentized Range (HSD) test. Columns followed by the same letter are not statistically different at P > 0.05.

The intensities of GFP expression mediated by the soybean promoters and a CaMV35S promoter construct in soybean hairy roots were determined using image analysis. Many of the Gmubi promoters gave rise to significantly higher levels of GFP expression than the CaMV35S promoter used as a control (ANOVA, P > 0.0001, Figure 5a). The strongest Gmubi promoters (Gmubi1, Gmubi2, Gmubi3, Gmubi4, Gmubi7 and Gmubi9) showed a ~2-4-fold increase in GFP expression over levels given by the CaMV35S promoter (Figure 5a). The Gmubi5, Gmubi6 and Gmubi8 promoters gave rise to similar or slightly higher levels of GFP than the CaMV35S promoter, while the Gmubi10 promoter showed the lowest GFP expression among the Gmubi promoters.

The GmERF promoters displayed somewhat lower GFP intensities in hairy roots than the Gmubi promoters but some of these promoters displayed higher expression levels than the CaMV35 promoter (ANOVA, P > 0.0001, Figure 5b). The GmERF2, GmERF6 and GmERF10 promoters showed ~1.4-1.7-times higher GFP than CaMV35S (Figure 5b). The GmERF3, GmERF4 and GmERF7 promoters exhibited similar GFP compared to the CaMV35S promoter; whereas the GmERF1, GmERF5, GmERF8 and GmERF9 promoters directed lower levels of GFP compared to the CaMV35S promoter.

**Figure 6:**
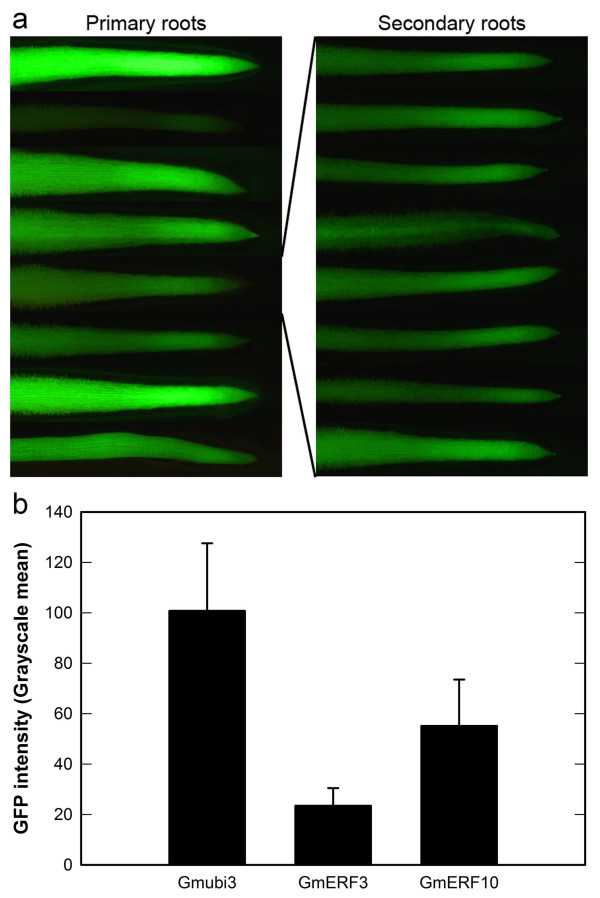
**GFP expression variation among independent hairy root events and within events**. (a) Primary roots containing the GmERF10 promoter and secondary hairy roots generated from a single GmERF10-containing primary root. (b) GFP intensity quantification in secondary hairy roots generated from single primary roots containing either the Gmubi3, GmERF3 or GmERF10 promoter. Values are means ± SD

Although the average GFP expression levels were determined based on image analysis of different soybean hairy root events, an unexpected large variation in GFP intensities among different root events was observed for all of the promoters evaluated, including the CaMV35S promoter. As a result of this large variation, the standard deviations for GFP intensity means were quite large for most of the promoters (Figure 5a-b). Analysis of GFP expression in numerous secondary roots from the same primary root event was performed using events containing the Gmubi3, GmERF3 and GmERF10 promoters. Although a large variation in GFP intensities was seen among primary roots, the variation in GFP intensity was much smaller in the secondary roots generated from single primary roots (Figure 6a). A remarkable reduction of the standard deviations for GFP intensity means in secondary roots was also apparent (Figure 6b).

### Southern hybridization analysis

**Figure 7:**
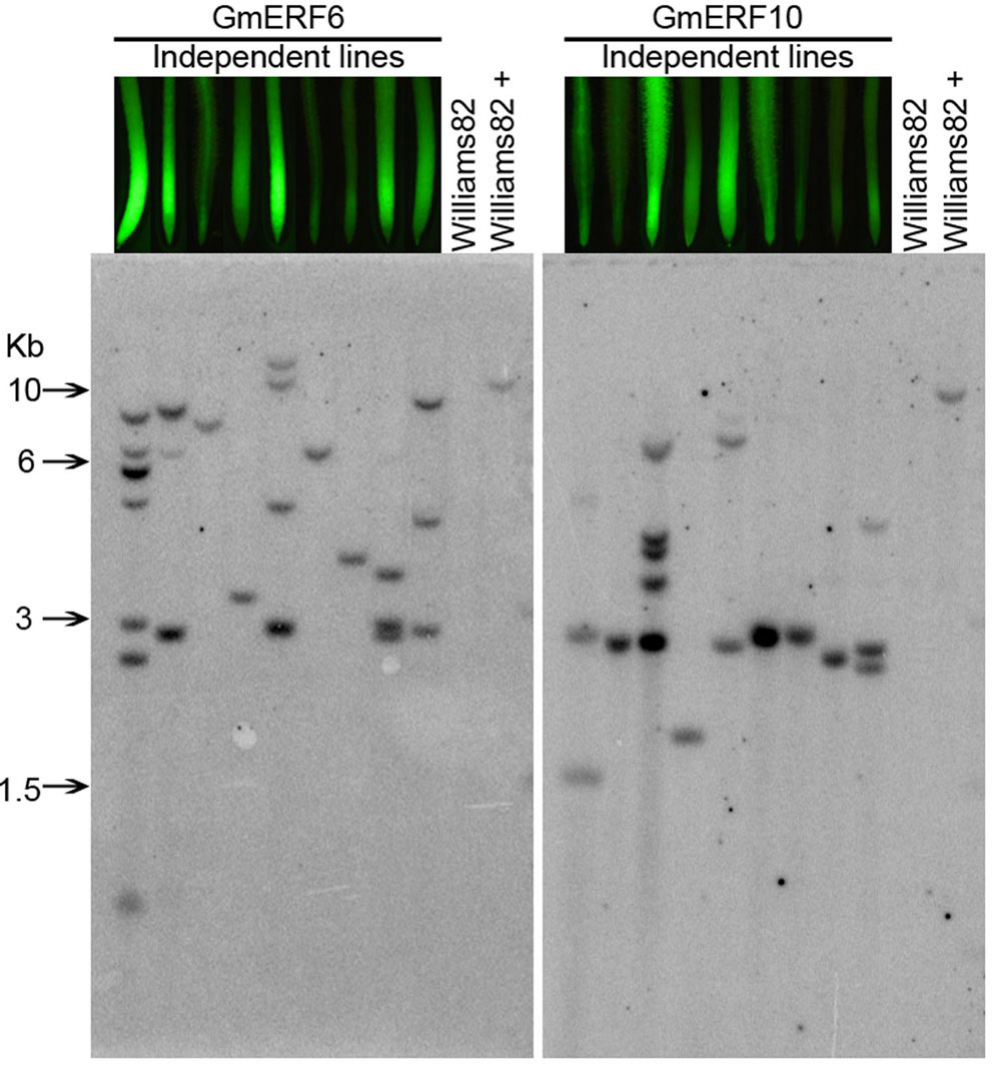
**Southern blot analysis of genomic DNA from hairy roots**. Images of root tips on top of lanes correspond to independent root events used for DNA extraction. DNA was digested with *Bsr*GI and hybridized with a ^32^P-labeled *gfp *probe. "Williams82" is DNA from roots induced with *A. rhizogenes *harboring no promoter construct, "Williams82 +" is Williams82 DNA plus 10 pg of respective plasmid, representing a single transgene copy in the soybean genome.

With the aim of studying the transgene integration patterns present in the soybean transgenic hairy roots and determining if a relationship existed between GFP intensity and transgene copy number, Southern hybridization analysis was conducted on genomic DNA isolated from either GmERF6- or GmERF10-containing hairy roots using the *gfp *coding region as the hybridization probe. Hybridization signals were detected in all the transformed hairy root lines analyzed (Figure 7), confirming the stable integration of the *gfp *coding sequence in the genomes. The lanes containing DNA from hairy roots induced with *A. rhizogenes *harboring no binary vector, showed no hybridization bands (lanes: *Williams82*, Figure 7). As *Bsr*GI recognizes a single site within the T-DNA of pCAMBIA-promoter constructs, the presence of one to seven variable-size bands in the genomic DNA from either GmERF6- or GmERF10-containing roots indicates T-DNA integrations ranging from one to seven copies (Figure 7).

Transgene copy number from the southern hybridization analysis was also directly correlated with the GFP expression intensity displayed in the transgenic hairy roots that were used for genomic DNA extraction (Additional file 4). Hairy root events with high transgene copy number generally displayed high GFP intensities; whereas, hairy roots events with single or low T-DNA copy number gave low or moderate GFP intensities.

## Discussion

### Bioinformatics analysis of the *Gmubi *and *GmERF *gene families of soybean

The polyubiquitin gene family in soybean (Figure 1A) contains three moderately well characterized genes (*Gmubi1, Gmubi2 *and *Gmubi3*); however, other family members have received little to no attention. The promoters regulating these genes have likewise not been well characterized but show promise as strong constitutive promoters based on recently-reported transcriptome data [45,46] and previous characterizations of a soybean polyubiquitin promoter (Gmubi) [7,10] that was recloned in this current research as a slightly longer promoter and renamed "Gmubi3".

The *ERF *genes were classified based on their coding sequences, and particularly on the presence of the well-conserved AP2/ERF DNA-binding domain [19]. The phylogeny of *GmERF *genes in this study (Figure 1b) was very similar to phylogenies previously reported for *ERF*s in rice, *Arabidopsis *and tobacco [19,42,47], confirming that this family of transcription factor is quite conserved among different plants. A previous phylogenetic analysis of *GmERF *genes revealed the presence of 98 unigenes containing a complete AP2/ERF domain in soybean [47]; however, we here report 359 AP2/ERF-containing *GmERF *genes using data from the recently released soybean genome assembly, representing a significant update for this gene family in soybean.

### Transient expression assays in soybean and lima bean cotyledons

Quantitative characterization of soybean promoters was rapidly assessed using both transient gene expression in lima bean cotyledons and stable expression in soybean hairy roots. We have previously reported the use of lima bean cotyledons for rapid analyses of transient gene expression [10] and characterization of viral suppressors of gene silencing [43,48]. In this report, we also evaluate soybean cotyledons as a potential target tissue for rapid validation of soybean promoters. In soybean cotyledons, initial attempts to visualize GFP at the 24 hour time point, which is the peak expression time for the lima bean target [10], were unsuccessful as only very low levels of GFP were observed. However, use of our automated image collection system [44] for semi-continuous monitoring of GFP expression revealed that the GFP protein apparently diffused rapidly from the initial target cell in soybean cotyledons, leading to depletion of scorable GFP levels (Figure 2, Additional file 3). In lima bean cells, rapid diffusion of GFP was not detected in the cells surrounding the original targeted cell, although it may occur at reduced levels. The loss of the GFP protein observed using soybean cotyledons suggests that there are basic differences in the epidermal cell structures in lima beans and soybeans. Confocal microscopy indeed confirmed some major differences in the anatomy of epidermal cells (Figure 3).

Retention of GFP in the targeted cells after bombardment is definitely preferable for gene expression analysis. The rapid loss of GFP in soybean cotyledonary cells made analysis difficult and this target tissue is completely unsuitable for transient expression analysis using single time point determinations. The presence of small amounts of GFP at the 24 h time point could be misinterpreted as the absence of expression, which was not the case. Since single time point determinations at 24 h are often used for transient expression analysis using GFP [33] and GUS [49], loss of transient gene expression as reported here in soybean cotyledonary tissues should be recognized as a potential problem in interpreting results. The use of dynamic semi-continuous monitoring of gene expression using our automated image collection system facilitated the detection of GFP loss from targeted cells, and movement into the surrounding cells. Without semi-continuous monitoring, movement of GFP may not have been perceived.

Transient expression of GFP in cells of lima bean cotyledons was far more consistent over time compared to soybean cotyledons (Figure 2). Lima bean cotyledons therefore offer a more suitable target tissue for quantitative transient GFP expression assays. Loss of GFP from the targeted soybean cotyledonary cells was somewhat reduced through the use of a translational fusion of GFP to the hygromycin resistance gene (Figure 2, 3), resulting in production of a larger fusion protein. However, use of this translational fusion resulted in much lower apparent GFP intensities and fewer foci (Figure 2 lower panels). We have previously reported that translational fusions containing GFP give rise to considerable reductions of transient GFP intensities in lima bean cotyledonary cells, probably due to either a quenching of fluorescence by the protein partner or conformational changes in GFP as a result of an alteration of the chromophore structure [43,48]. Although use of translational fusions can be used to minimize loss of the small GFP protein from certain target tissues, the effects of the fusion partner on GFP detection need to be considered when this approach is utilized.

### Transient expression mediated by Gmubi and GmERF promoters

In this study, the Gmubi1-9 promoters were isolated from polyubiquitin genes sharing high homology (Figure 1a) but containing variable numbers of the ubiquitin-coding unit [50]. The *Gmubi1*, *Gmubi2*, *Gmubi3 *and *Gmubi8 *contained 4 ubiquitin-coding units; the *Gmubi4 *and *Gmubi6 *contained 7 ubiquitin-coding units; and the *Gmubi5*, *Gmubi7 *and *Gmubi9 *contained 6, 5 and 2 ubiquitin-coding units, respectively. The Gmubi10 promoter was isolated from a more distant relative gene containing a monomeric ubiquitin-coding unit (Figure 1a). Although the Gmubi1-9 promoters gave rise to relatively high levels of gene expression, the Gmubi10 promoter displayed consistently low expression levels in both transient expression and hairy roots. All of the reports to date describing ubiquitin promoters in different plants have focused on polyubiquitin gene promoters [10,15,51].

The Gmubi promoters characterized here were either intron-containing or intron-less promoters. The *Gmubi1-7 *gene sequences contained predicted introns in the 5'-UTR, which were predicted to splice to acceptor sites generated during promoter cloning just prior to the initiation codon of the *gfp *coding sequence. The *Gmubi8-10 *gene sequences contained no predicted introns in the 5'-UTR. To our knowledge, no characterization of native intron-less plant ubiquitin promoters has been previously reported. Although in this study there were no evident differences between transient GFP expression levels mediated by the intron-containing or the intron-less Gmubi promoters, the introns within the 5'UTR of most polyubiquitin promoters quantitatively enhance transgene expression levels [51,52].

Although most of the Gmubi promoters directed overall high expression levels, the Gmubi3 promoter gave exceptionally high levels of GFP expression. This high gene expression driven by the Gmubi3 promoter is not surprising as the *Gmubi3 *gene is highly active in different organs of soybean [45,46]. We previously reported 5-fold greater transient GFP expression using a slightly truncated version of the Gmubi3 promoter (917 bp; Gmubi) compared to a CaMV35S promoter [10]. In the present study, the Gmubi3 promoter (1438 bp) gave rise to 7-fold greater transient GFP expression compared to the same CaMV35S promoter. We have also reported that removal of the intron from the 5'UTR of the Gmubi promoter resulted in much lower levels of both transient expression in lima bean cotyledons [10] and stable expression in transgenic soybeans [7]. Although the intensity of expression was altered by the removal of the intron from the 5'UTR of the Gmubi promoter, the pattern of expression remained the same. Collectively, these results indicate that the intronic and upstream regions of this promoter may contain important *cis*-regulatory elements responsible for high levels of expression. An in-depth functional analysis of the Gmubi3 promoter may allow the identification of specific promoter elements that lead to this high gene expression.

The transient expression profiles from the GmERF promoters (Figure 4) were similar regardless of promoter strength. However, the time of peak GFP expression for the different GmERF promoters was more inconsistent compared to the expression peaks for the Gmubi promoters. This variability in expression peaks among the GmERF promoters may be associated with the transcriptional regulation of *ERF *genes under conditions of stress [22,23].

The GmERF promoters characterized in the present study were isolated from group IX *GmERF *genes (Figure 1b), which share high homology with group IX tobacco *ERF *genes [42]. Transcription of group IX *ERF *genes in tobacco and soybean can be induced after wounding or exogenous application of methyl jasmonate (MeJa) [42,47]. Particularly, a group IX soybean *ERF *gene (GmERF69), with high similarity to the *GmERF10 *gene sequence identified here (Table 2), displayed high expression levels in soybean seedlings after exogenous application of MeJa, ethylene and salicylic acid, or growth under cold and salt conditions [47]. As gene transcription is largely regulated by *cis*-acting regulatory elements within the promoter sequences [53], the promoters from the group IX *GmERF *genes may be good candidates to direct inducible transgene expression. Further in-depth functional characterization of the GmERF promoters validated herein along with their potential *cis*-regulatory elements may also be of particular interest to increase the current knowledge of gene transcription regulation under various stress conditions.

**Table 2:** Gene IDs for *Gmubi *and *GmERF *genes and the respective sizes of their isolated promoters

Gmubi promoters			GmERF promoters		
**Promoter**	**Size (bp)**	**Gene ID**	**Promoter**	**Size (bp)**	**Gene ID**

Gmubi1	1449	Glyma10g39780	GmERF1	1171	Glyma20g16920.1
Gmubi2	1484	Glyma13g17830.1	GmERF2	1243	Glyma20g16910.1
Gmubi3	1438	Glyma20g27950.1	GmERF3	1331	Glyma11g03900.1
Gmubi4	1430	Glyma13g24470.1	GmERF4	1137	Glyma01g41530.1
Gmubi5	1452	Glyma13g24500.1	GmERF5	1166	Glyma05g05180.1
Gmubi6	1409	Glyma07g32020.1	GmERF6	1257	Glyma05g05130.1
Gmubi7	1453	Glyma17g04690.1	GmERF7	1310	Glyma19g43820.1
Gmubi8^1^	892	Glyma10g05830.1	GmERF8	967	Glyma20g34570.1
Gmubi9	1314	Glyma13g20200	GmERF9	1125	Glyma10g33060.1
Gmubi10^2^	1355	Glyma15g13650.1	GmERF10	1196	Glyma17g15460.1

^1^The Gmubi8-10 promoters do not contain intron sequences within their 5'UTR.

^2^The Gmubi10 promoter was isolated from a monoubiquitin gene containing a monomeric ubiquitin-coding unit.

### Gene expression mediated by Gmubi and GmERF promoters in hairy roots

The percentage of GFP-positive hairy roots achieved here (72%, Table 1) is substantially higher than previously reported for *A. rhizogenes*-induced hairy roots of soybean (50%) [38]. The development of the hairy root phenotype caused by *A. rhizogenes *is the result of the integration and expression of T-DNA contained in the bacterial root inducing (Ri) plasmid in the plant genome [54]. *A. rhizogenes *can also transfer the T-DNA from binary vectors, leading to the formation of hairy roots with or without the binary vector T-DNA. The ratios of hairy roots with and without the binary vector T-DNA can vary tremendously across different plants [38].

GFP detection and analysis in hairy roots was relatively straightforward as hairy roots do not contain chlorophyll, which can otherwise interfere with GFP detection [55]. To counteract chlorophyll interference with GFP detection, different methodologies have been developed for chlorophyll elimination in photosynthetic tissues, including exposure to alcohol [56], application of photobleaching herbicides [57] or use of gene silencing to suppress the *Phytoene desaturase *(*PDS*) gene [58]. However, chlorophyll elimination treatments are notably harsh and demand additional manipulation of tissues, which may alter transgene expression, particularly expression of inducible DNA constructs.

GFP expression in soybean hairy roots was quite variable among different events containing the same promoter construct, although some general conclusions could be made about promoter strength using this validation tool. Similar to quantitative analysis of transient expression in lima bean cotyledonary tissue, the Gmubi3 and the Gmubi10 promoters also gave the highest and the lowest GFP intensity, respectively, in soybean hairy roots. The Gmubi1, Gmubi4, Gmubi7, Gmubi8 and Gmubi9 promoters showed quite similar GFP expression intensities using both validation tools. However, a comparison of transient and stable expression intensities using the Gmubi2, Gmubi5 and Gmubi6 promoters showed some inconsistencies in promoter strength using the two different validation tools (moderate, high and high transient expression but high, moderate and moderate expression in stably-transformed hairy roots, respectively;Table 3). The Gmubi2, Gmubi5 and Gmubi6 promoters may be the most interesting for further analysis as they show the greatest disparity in expression using transient and stable expression analyses.

**Table 3:** Grouping of the Gmubi and GmERF promoters based on the CaMV35S-driven GFP expression.

	Transient GFP expression			GFP expression in hairy roots		
**Promoter**	**Low**	**Moderate**	**High**	**Low**	**Moderate**	**High**

Gmubi #	10	2, 8	1, 3, 4, 5, 6, 7, 9	10	5, 6, 8	1, 2, 3, 4, 7, 9
GmERF #	1, 8, 9	2, 4, 7	3, 5, 6, 10	1, 5, 8, 9	3, 4, 7	2, 6, 10

GFP levels driven by the CaMV35S promoter in both transient and hairy roots were considered as moderate

The GFP intensities determined for GmERF promoters using hairy roots in general also correlated with the transient GFP levels determined using lima bean cotyledons. The GmERF2, GmERF3 and GmERF5 promoters were the most inconsistent expressers in this group (moderate, high and high transient GFP expression, but high, moderate and low GFP intensities in hairy roots, respectively; Table 3). Expression directed by these promoters may be affected by the wounding or other stresses caused by tissue manipulation and particle bombardment. Further studies on these 3 promoters in stably-transformed tissues may be of particular interest to identify regulatory regions within promoters that are responsive to various stimuli.

The transient expression system reported here differs considerably from the hairy root expression system, relative to the fate of the introduced DNA and the nature of the expressing tissue. Any consistency in expression intensity using the two validation tools, suggests a certain robustness in promoter activity. For transient expression using particle bombardment, large amounts of DNA are introduced [59] on each particle and cells that express the introduced DNA usually contain a particle in, or adjacent to the nucleus [60,61]. Transient expression results in a rapid increase in gene expression, followed by a rapid decline (Figure 4), which has been partly attributed to gene silencing of transient expression [43,48]. Therefore, during transient expression in lima bean cotyledonary cells, large amounts of plasmid DNA are delivered to the nucleus, which result in very high levels of extrachromosomal gene expression. Preintegrative, extrachromosomal DNAs may not be subject to the same regulatory influences as genomic DNA and this DNA may have different access to transcription factors. Nevertheless, transient expression might be a good early indicator of promoter strength in stably-transformed tissues [7].

Stably-expressed promoters that are introduced in soybean hairy roots are integrated into genomic DNA and expression in this tissue may more accurately reflect promoter activity in its native context. However, gene expression may also be affected by integration site and transgene copy number [62], as well as the status of the transgenic tissues. Although the soybean hairy root system may not be optimal for validation of some tissue-specific promoters, we have successfully used this system for validation of large number of promoters including promoters identified as "seed specific" (data not shown). Consistency in the intensity of gene expression using these two different validation tools suggests good stability and accurate prediction of relative promoter strengths.

### Southern hybridization analysis and transgene copy number

GFP intensities were quite variable in independent primary hairy root events (Figure 6). This variation in gene expression across stably-transformed events has been often attributed to the site(s) of transgene insertion and transgene copy number [5,34]. The insertion site, copy number and structure of integrated DNA differs, depending on the transformation methods utilized. Direct transformation methods such as particle bombardment can frequently result in the insertion of large copy numbers of plasmid DNA at a single-site, leading to transgene silencing [63,64]. Gene cassettes or minimal constructs can reduce or eliminate this effect [65,66]. On the other hand, transformation using *Agrobacterium *typically results in lower copy number gene introductions, which has been reported to give more consistent transgene expression [63,64].

Our results suggest that the variability in *gfp *gene expression in soybean hairy roots was associated with the copy number of the introduced T-DNA. The highest GFP expression levels were associated with roots that contained the highest copy numbers of introduced DNAs. Use of *Agrobacterium tumefaciens *for transformation usually results in the integration of single or low T-DNA copies into the plant genome [67,68]. Although use of *A. rhizogenes *can lead to high copy T-DNA integration [35,36,38], the relationship between high copy number integration and transgene expression has not been previously reported in hairy roots. Using *Arabidopsis *plants containing sequentially increasing copy numbers of a CaMV35S-driven *gfp *gene, Schubert et al. [62] demonstrated increases in GFP expression levels when up to 4 copies of a CaMV35S-driven *gfp *gene were present. As the copy number was increased to 5 and greater, GFP expression was suppressed. Schubert et al. [62] further suggested that suppression occurs once a gene expression threshold is reached and is gene-specific.

In this study, hairy roots containing up to 7 T-DNA inserts (Figure 7) displayed the highest GFP expression and did not show gene suppression. A significant correlation of high GFP expression with high copy number integration was observed with the GmERF6 and GmERF10 promoters (Additional file 4), both of which displayed higher expression levels than the CaMV35S promoter in soybean hairy roots (Figure 5b). If a threshold copy number/expression level is required to silence the *gfp *gene, that threshold was not reached in the transgenic hairy roots.

The use of hairy roots to validate promoter activity is a simple alternative for gauging promoter strength in stably-transformed plants, although the influence of copy number on gene expression should be considered [69]. The transient expression analysis used in this research may nevertheless be more reflective of general promoter strength as each cell receives similar high copy numbers of each DNA construct, and hundreds to thousands of cells are collectively analyzed. As transient expression is analyzed prior to DNA integration, complications from conformational and positional effects in genomic DNA are avoided.

## Conclusions

We report here the isolation and characterization of 20 novel soybean promoters from two different gene families, *ubiquitin *and *ERF*. A rapid quantitative evaluation of promoter strength was consistently performed in both transiently-expressing cotyledonary tissues of lima bean and stably-transformed hairy roots of soybean. We also provide novel insights towards the utilization of transient and stable expression systems for promoter validation.

## Methods

### Phylogenetic analysis of the *Gmubi *and *GmERF *genes

The *ubiquitin *genes were identified in the soybean genome assembly (accessed in April, 2009; ftp://ftp.jgi-psf.org/pub/JGI_data/Glycine_max/Glyma1/annotation/) based on the presence of the highly conserved ubiquitin-coding unit. The soybean *ERF/AP2 *genes were obtained from SoyDB: A Knowledge Database of Soybean Transcription Factors (http://casp.rnet.missouri.edu/soydb/) and verified using the Soybean Transcription Factor Knowledge Base (http://www.igece.org/Soybean_TF/).

The phylogenetic trees for *Gmubi *and *GmERF *gene families were constructed with the aligned amino acid sequences using MEGA 4.0 [70] and the Neighbor-Joining (NJ) method [71]. For each gene family, the bootstrap consensus tree was inferred from 1000 replicates [72] and drawn to scale, with branch lengths in the same units as those of the evolutionary distances used to infer the phylogenetic tree. The evolutionary distances were computed using the Poisson correction method [73] and are in the units of the number of amino acid substitutions per site.

### DNA constructs

**Figure 8:**
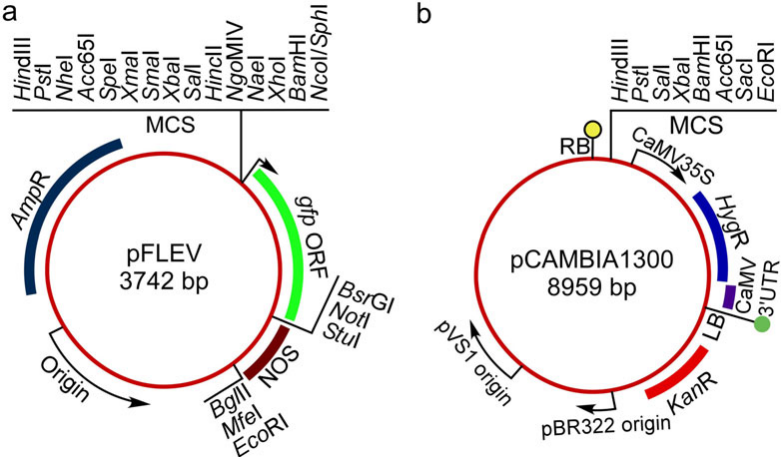
**Expression vectors used for promoter cloning and functional assays**. (a) Engineered PUC19-derived expression vector (pFLEV; Finer Laboratory Expression Vector), containing unique restriction sites for promoter cloning and manipulation. (b) pCAMBIA1300 vector used for hairy root production. *MCS *multiple cloning site, *RB *right border, *LB *left border.

For rapid direct cloning and subcloning of soybean promoters, a PUC19-derived expression vector (pFLEV; Finer Laboratory Expression Vector) was generated (Figure 8a). For construction of pFLEV, a synthetic multiple cloning site (MCS) containing unique restriction sites and flanked by *Hin*dIII and either *Nco*I or *Sph*I restriction sites was designed and introduced 5' to a *gfp *gene encoding a soluble GFP [74]. The *gfp *gene was succeeded by a nopaline synthase terminator (NOS) sequence. Additional restriction sites located at the 3' end of the *gfp *sequence (*Bsr*GI, *Not*I, *Stu*I) and at the 3' end of NOS (*Bgl*II, *Mfe*I, *Eco*RI) were included in pFLEV to allow further mobilization of expression cassettes into different expression vectors.

The DNA sequences lying immediately upstream of the coding regions of 20 selected *Gmubi *and *GmERF *genes (Table 2) were PCR-amplified using specific primers (Additional file 5). Intronic regions (5' UTR) present in the coding sequences of *Gmubi*1-7 were also included in the 3' end of their respective cloned promoters. PCR-amplifications were conducted on genomic DNA from soybean (*G. max *'Jack') using the FailSafe™ PCR Kit (EPICENTRE^® ^Biotechnologies, Madison, WI, USA). PCR products were purified, digested and inserted into the MCS of pFLEV (Figure 8a). All sequences of cloned promoters were confirmed by DNA sequencing. Promoter-containing pFLEV constructs were used for transient expression analysis in lima bean (*P. lunatus *'Henderson-Bush') cotyledons.

For construction of the binary version of the promoter constructs, the complete expression cassettes composed of promoter, *gfp *coding sequence and NOS terminator were excised from pFLEV using appropriate restriction enzymes and cloned into the MCS of appropriately digested pCAMBIA1300 (CAMBIA, Canberra, Australia; Figure 8b). For soybean hairy root production, pCAMBIA1300-promoter constructs were introduced into *A. rhizogenes *strain K599 (kindly provided by Dr. Harold Trick, Kansas State University) by the freeze-thaw method [75].

### Transient expression analysis

Soybean (*G. max *'Jack') seeds were harvested from plants grown in the greenhouse (16/8 h light:dark, 28°C) with supplemental lighting from high pressure sodium lamps. Lima bean seeds were harvested from plants grown in a growth chamber (50% relative humidity, 16/8 h light:dark, 25/23°C day/night). Both soybean and lima bean seeds were surface sterilized in a 10% (v/v) bleach solution with slow agitation for 20 min, rinsed 4-7 times with sterile water and germinated between moistened sterile paper towels contained in GA7 culture vessels.

Transient expression was initially compared in soybean and lima bean cotyledons using a 35S-driven GFP construct [10] and a 35S-driven GFP::Hygromycin gene fusion (GFP::Hygromycin) [43]. Soybean and lima bean cotyledons were excised from 2-d-old and 4-d-old germinating seedlings, respectively. DNA constructs were precipitated onto tungsten particles and introduced into the adaxial surface of the cotyledons utilizing a Particle Inflow Gun [76]. Bombarded cotyledons were placed adaxial side up on OMS culture medium containing MS salts [77], B5 vitamins [78], 3% sucrose and 0.2% Gelrite (pH 5.7) for GFP monitoring. Semi-continuous image acquisition was performed using an automated image collection system [8] composed of a MZFLIII dissecting microscope (Leica, Heerbrugg, Switzerland) equipped with a "GFP-2" filter set (Excitation 480 ± 40 nm, Emission 510 nm), a Spot-RT CCD digital camera (Diagnostic Instruments Inc., Sterling Heights, MI, USA) and a robotics platform (Arrick Robotics Inc., Hurst, TX, USA). Soybean and lima bean cotyledons showing transient GFP expression were also examined 10 h post bombardment using a Leica TCS SP5 II confocal laser microscope (Leica, Heerbrugg, Switzerland). Based on the more consistent GFP expression patterns obtained using lima bean cotyledons, transient expression analysis of all 20 different cloned soybean promoters was conducted using lima bean cotyledons as the target tissue.

Quantitative analysis of transient GFP expression directed by the 20 novel soybean promoters in lima bean cotyledons was performed as previously described [10,44]. GFP expression levels for each promoter were calculated and presented as the percentage of the peak GFP expression of the CaMV35S promoter. For each promoter construct, 5 to 9 cotyledons were bombarded and monitored for 100 h, over at least two independent experiments.

### Hairy root induction and analysis

For induction of soybean hairy roots, cotyledons were inoculated as previously described [35] with some modifications. *A. rhizogenes *harboring the pCAMBIA1300-promoter constructs was grown overnight in 2 ml liquid YEP (Yeast Extract Peptone) medium containing 100 mg l^-1 ^kanamycin. *A. rhizogenes *without the binary vector was grown in YEP medium lacking antibiotics. Soybean (*G. max *'Williams82') seeds were surface-sterilized and germinated in GA7 containers as described above. After 5 d, cotyledons were excised and wounded several times on the abaxial side with a sterile scalpel dipped in the bacterial cultures. Inoculated cotyledons were cultured abaxial side up on P5 Fisherbrand^® ^(Fisher Scientific, Pittsburgh, PA, USA) filter paper moistened with sterile distilled water. After 3 d, cotyledons were transferred to OMS medium containing 400 mg l^-1 ^Timentin for hairy root induction. Cotyledons were incubated at 25°C with a 16:8 h light:dark photoperiod under an illumination of 40 μEm^-2^s^-1^.

GFP-expressing hairy roots (~2 cm) were excised from cotyledons and subcultured for 4 d on OMS medium containing 400 mg l^-1 ^Timentin. Root tip regions were imaged utilizing the same microscope and camera used previously for transient GFP detection but the robotics components were disabled. Image analysis of roots was performed using ImageJ software [79]. High-resolution images (1600 × 1200 pixels) of individual root tips, ~5 mm in length, were separated into red, blue and green channels and only the green channel data was used for quantification of GFP intensity. Due to the reflection of fluorescence through the culture medium next to GFP-expressing roots, the background gray value of a 100 × 100 pixel area adjacent to each root was first subtracted from every pixel present in this channel. The threshold levels were then adjusted to segment the expressing pixels from root images and the grayscale mean value of the background-corrected channel was then determined. An average grayscale mean value from the slight background fluorescence in the green channel from hairy roots induced with *A. rhizogenes *without the binary vector was also determined. GFP intensity for each root was calculated by subtracting the average grayscale means of roots induced with *A. rhizogenes *containing no binary vector from the grayscale means of the transgenic GFP-expressing hairy roots using the green channel. For each promoter construct, 14 to 32 independent hairy root events were analyzed, over at least two independent experiments. Statistical analysis was performed using SAS 9.2 TS (SAS Institute Inc., Cary, NC, USA).

### Southern hybridization analysis

Southern blot analysis was performed using genomic DNA isolated from 18 transformed hairy root events containing the *gfp *gene regulated by either the GmERF6 or GmERF10 promoter. Genomic DNA was extracted from lyophilized root tissues according to Murray and Thompson [80] as modified by Fulton et al. [81]. DNAs from each independent root event (10 μg) were digested overnight with *Bsr*GI, which cuts the T-DNA harboring the GmERF6 or GmERF10 promoter at a single site, only 10 bp from the 3' end of the *gfp *gene. Digested DNAs were separated on 0.8% (w/v) agarose gels and then transferred to nylon membranes (Roche Diagnostics GmbH, Indianapolis, IN, USA) as described by Sambrook et al. [82]. The hybridization probe was a 717 bp fragment of the *gfp *coding region amplified by PCR using the primers 5'ATGGTGAGCAAGGGCGAGGAGCTG3' and 5'TTACTTGTACAGCTCGTCCATG3'. The probe was labeled with [α-^32^P]-dCTP (Perkin-Elmer, Boston, MA, USA) using the Prime-It^® ^II Random Labeling Kit (Stratagene, La Jolla, CA, USA) according to the manufacturer's instructions. The labeled probe was hybridized to the membranes and incubated overnight at 60°C. The hybridized membranes were exposed to a phosphor screen holder for 24 h and then scanned with a Storm 860 PhosphorImager™ System (Molecular Dynamics, Sunnyvale, CA, USA) for visualization of hybridization patterns.

## List of abbreviations

CaMV35S: Cauliflower Mosaic Virus 35S; MS: Murashige and Skoog; OMS: MS medium containing no plant growth regulators; ERF: Ethylene Response Factor; Gmubi: *Glycine max *Ubiquitin; GmERF: *Glycine max *Ethylene Response Factor; 5' UTR: 5' Untranslated Region; pFLEV: Finer Laboratory Expression Vector; MCS: Multiple Cloning Site; PCR: Polymerase Chain Reaction.

## Authors' contributions

CMHG cloned the GmERF promoters, developed and optimized the transient expression and hairy root validation tools, designed experiments, collected and analyzed data for GmERF promoters, and drafted the manuscript. RAB cloned the Gmubi promoters and collected data for Gmubi-mediated expression in transient expression and hairy roots. PJR performed the phylogenetic analysis of *Gmubi *and *GmERF *gene families. MLJ contributed to the interpretation of gene expression analysis in hairy roots and critically revised the manuscript. XC identified the *Gmubi *and *GmERF *gene families in the soybean genome and transcription factor databases. MPT assisted with Gmubi promoter identification and critically revised the manuscript. JJF was the principal investigator, conceived of the experiments and contributed to project design, isolation of the Gmubi promoters, data analysis, and manuscript drafting. All authors read and approved the final manuscript.

## Supplementary Material

Additional file 1 **List of predicted amino acid sequences used for the phylogenetic analysis of the *Gmubi *genes**. The *Gmubi *genes were identified in the soybean genome assembly (accessed in April, 2009; ftp://ftp.jgi-psf.org/pub/JGI_data/Glycine_max/Glyma1/annotation/) based on the presence of the highly conserved ubiquitin-coding unit. Click here for file

Additional file 2 **List of AP2 domains used for the phylogenetic analysis of the *GmERF *genes**. The soybean *GmERF *genes were obtained from SoyDB: A Knowledge Database of Soybean Transcription Factors (http://casp.rnet.missouri.edu/soydb/) and verified using the Soybean Transcription Factor Knowledge Base (http://www.igece.org/Soybean_TF/). Click here for file

Additional file 3 **A time-lapse animation of soybean and lima bean cotyledons transformed with the 35S-GFP construct**. Images were collected every hour for 100 h using an automated image collection system and assembled using ImageReady. Click here for file

Additional file 4 **Regression analysis of GFP expression and transgene copy number scored on the Southern blots**. GFP expression for each hairy root shown on Figure 7 was quantified and grayscale values correlated with the respective transgene copy number. The regression analysis showed P-Values of 0.005 and 0.039 for GmERF6- and GmERF10-containing hairy roots, respectively. Click here for file

Additional file 5 **List of primer sequences used to PCR-amplify the soybean promoters**. Restriction sites incorporated in the forward and reverse primers are underlined. *F *forward primer, *R *reverse primer. Click here for file
