# Supplementary material for: High level transgenic expression of soybean (Glycine max) GmERF and Gmubi gene promoters isolated by a novel promoter analysis pipeline
Source: BMC Plant Biol. 2010 Nov 4;10:237. doi: 10.1186/1471-2229-10-237 (PMC3095320; doi:10.1186/1471-2229-10-237)
Supplement: Additional file 1 — List of predicted amino acid sequences used for the phylogenetic analysis of the Gmubi genes. The Gmubi genes were identified in the soybean genome assembly (accessed in April, 2009; ftp://ftp.jgi-psf.org/pub/JGI_data/Glycine_max/Glyma1/annotation/) based on the presence of the highly conserved ubiquitin-coding unit. [file 1471-2229-10-237-S1.PDF]

Additional file 1. List of predicted amino acid sequences used for the phylogenetic analysis of the *Gmubi* genes. The *Gmubi* genes were identified in the soybean genome assembly (accessed in April, 2009; ftp://ftp.jgi-psf.org/pub/JGI_data/Glycine_max/Glyma1/annotation/) based on the presence of the highly conserved ubiquitin-coding unit.

> Glyma10g39780 (Gmubi1)

MQIFVKTLTGKTITLEVESSDTIDNVKAKIQDKEGIPPDQQRLIFAGKQLEDGRTLADYNIQKESTLHLVLRL
RGGMQIFVKTLTGKTITLEVESSDTIDNVKAKIQDKEGIPPDQQRLIFAGKQLEDGRTLADYNIQKESTLHLV
LRLRGGMQIFVKTLTGKTITLEVESSDTIDNVKAKIQDKEGIPPDQQRLIFAGKQLEDGRTLADYNIQKESTL
HLVLRLRGGMQIFVKTLTGKTITLEVESSDTIDNVKAKIQDKEGIPPDQQRLIFAGKQLEDGRTLADYNIQK
ESTLHLVLRLRGGF

>Glyma13g17830.1 (Gmubi2)

MQIFVKTLTGKTITLEVESSDTIDNVKAKIQDKEGIPPDQQRLIFAGKQLEDGRTLADYNIQKESTLHLVLRL
RGGMQIFVKTLTGKTITLEVESSDTIDNVKAKIQDKEGIPPDQQRLIFAGKQLEDGRTLADYNIQKESTLHLV
LRLRGGMQIFVKTLTGKTITLEVESSDTIDNVKAKIQDKEGIPPDQQRLIFAGKQLEDGRTLADYNIQKESTL
HLVLRLRGGMQIFVKTLTGKTITLEVESSDTIDNVKAKIQDKEGIPPDQQRLIFAGKQLEDGRTLADYNIQK
ESTLHLVLRLRGGF

>Glyma20g27950.1 (Gmubi3)

MQIFVKTLTGKTITLEVESSDTIDNVKAKIQDKEGIPPDQQRLIFAGKQLEDGRTLADYNIQKESTLHLVLRL
RGGMQIFVKTLTGKTITLEVESSDTIDNVKAKIQDKEGIPPDQQRLIFAGKQLEDGRTLADYNIQKESTLHLV
LRLRGGMQIFVKTLTGKTITLEVESSDTIDNVKAKIQDKEGIPPDQQRLIFAGKQLEDGRTLADYNIQKESTL
HLVLRLRGGMQIFVKTLTGKTITLEVESSDTIDNVKAKIQDKEGIPPDQQRLIFAGKQLEDGRTLADYNIQK
ESTLHLVLRLRGGF

>Glyma13g24470.1 (Gmubi4)

MQIFVKTLTGKTITLEVESSDTIDNVKSKIQDKEGIPPDQQRLIFAGKQLEDGRTLADYNIQKESTLHLVLRL
RGGMQIFVKTLTGKTITLEVESSDTIDNVKAKIQDKEGIPPDQQRLIFAGKQLEDGRTLADYNIQKESTLHLV
LRLRGGMQIFVKTLTGKTITLEVESSDTIDNVKAKIQDKEGIPPDQQRLIFAGKQLEDGRTLADYNIQKESTL
HLVLRLRGGMQIFVKTLTGKTITLEVESSDTIDNVKAKIQDKEGIPPDQQRLIFAGKQLEDGRTLADYNIQK
ESTLHLVLRLRGGMQIFVKTLTGKTITLEVESSDTIDNVKAKIQDKEGIPPDQQRLIFAGKQLEDGRTLADY
NIQKESTLHLVLRLRGGMQIFVKTLTGKTITLEVESSDTIDNVKAKIQDKEGIPPDQQRLIFAGKQLEDGRTL
ADYNIQKESTLHLVLRLRGGMQIFVKTLTGKTITLEVESSDTIDNVKAKIQDKEGIPPDQQRLIFAGKQLEDG
RTLADYNIQKESTLHLVLRLRGGF

>Glyma13g24500.1 (Gmubi5)

MQIFVKTLTGKTITLEVESSDTIDNVKSKIQDKEGIPPDQQRLIFAGKQLEDGRTLADYNIQKESTLHLVLRL
RGGMQIFVKTLTGKTITLEVESSDTIDNVKAKIQDKEGIPPDQQRLIFAGKQLEDGRTLADYNIQKESTLHLV
LRLRGGMQIFVKTLTGKTITLEVESSDTIDNVKAKIQDKEGIPPDQQRLIFAGKQLEDGRTLADYNIQKESTL
HLVLRLRGGMQIFVKTLTGKTITLEVESSDTIDNVKAKIQDKEGIPPDQQRLIFAGKQLEDGRTLADYNIQK
ESTLHLVLRLRGGMQIFVKTLTGKTITLEVESSDTIDNVKAKIQDKEGIPPDQQRLIFAGKQLEDGRTLADY
NIQKESTLHLVLRLRGGMQIFVKTLTGKTITLEVESSDTIDNVKAKIQDKEGIPPDQQRLIFAGKQLEDGRTL
ADYNIQKESTLHLVLRLRGGF

>Glyma07g32020.1 (Gmubi6)

MQIFVKTLTGKTITLEVESSDTIDNVKAKIQDKEGIPPDQQRLIFAGKQLEDGRTLADYNIQKESTLHLVLRL
RGGMQIFVKTLTGKTITLEVESSDTIDNVKAKIQDKEGIPPDQQRLIFAGKQLEDGRTLADYNIQKESTLHLV
LRLRGGMQIFVKTLTGKTITLEVESSDTIDNVKAKIQDKEGIPPDQQRLIFAGKQLEDGRTLADYNIQKESTL
HLVLRLRGGMQIFVKTLTGKTITLEVESSDTIDNVKAKIQDKEGIPPDQQRLIFAGKQLEDGRTLADYNIQK
ESTLHLVLRLRGGMQIFVKTLTGKTITLEVESSDTIDNVKAKIQDKEGIPPDQQRLIFAGKQLEDGRTLADY
NIQKESTLHLVLRLRGGMQIFVKTLTGKTITLEVESSDTIDNVKAKIQDKEGIPPDQQRLIFAGKQLEDGRTL
ADYNIQKESTLHLVLRLRGGMQIFVKTLTGKTITLEVESSDTIDNVKAKIQDKEGIPPDQQRLIFAGKQLEDG
RTLADYNIQKESTLHLVLRLRGGF

>Glyma17g04690.1 (Gmubi7)

MQIFVKTLTGKTITLEVESSDTIDNVKAKIQDKEGIPPDQQRLIFAGKQLEDGRTLADYNIQKESTLHLVLRL
RGGMQIFVKTLTGKTITLEVESSDTIDNVKAKIQDKEGIPPDQQRLIFAGKQLEDGRTLADYNIQKESTLHLV
LRLRGGMQIFVKTLTGKTITLEVESSDTIDNVKAKIQDKEGIPPDQQRLIFAGKQLEDGRTLADYNIQKESTL
HLVLRLRGGMQIFVKTLTGKTITLEVESSDTIDNVKAKIQDKEGIPPDQQRLIFAGKQLEDGRTLADYNIQK
ESTLHLVLRLRGGMQIFVKTLTGKTITLEVESSDTIDNVKAKIQDKEGIPPDQQRLIFAGKQLEDGRTLADY
NIQKESTLHLVLRLRGGC

> Glyma10g05830.1 (Gmubi8)

MQIFVKTLTGKTITLEVESSDTIDNVKAKIQDKEGIPPDQQRLIFAGKQLEDGRTLADYNIQKESTLHLVLRL
RGGMQIFVKTLTGKTITLEVESSDTIDNVKAKIQDKEGIPPDQQRLIFAGKQLEDGRTLADYNIQKESTLHLV
LRLRGGMQIFVKTLTGKTITLEVESSDTIDNVKAKIQDKEGIPPDQQRLIFAGKQLEDGRTLADYNIQKESTL
HLVLRLRGGMQIFVKTLTGKTITLEVESSDTIDNVKAKIQDKEGIPPDQQRLIFAGKQLEDGRTLADYNIQK
ESTLHLVLRLRGGDF

>Glyma13g20200 (Gmubi9)

MQIFVKTLTGKTITLEVESSDTIDNVKAKIQDKEGIPPDQQRLIFAGKQLEDGRTLADYNIQKESTLHLVLRL
RGGMQIFVKTLTGKTITLEVESSDTIDNVKAKIQDKEGIPPDQQRLIFAGKQLEDGRTLADYNIQKESTLHLV
LRLRGGDF

>Glyma15g13650.1 (Gmubi10)

MQIFVKTLTGKTITLEVESSDTIDNVKAKIQDKEGIPPDQQRLIFAGKQLEDGRTLADYNIQKESTLHLVLRL
RGGAKKRKKKTYTKPKKIKHKHKKVKLALLQFYKVDDSGKVQRLRKECPNAECGAGTFMANHFDRHYC
GKCGLTYVYQKAEA

>Glyma19g38170.1

MQIFVKTLTGKTITLEVESSDTIDNVKAKIQDKEGIPPDQQRLIFAGKQLEDGRTLADYNIQKESTLHLVLRL
RGGIIEPSLMALARKYNQDKMICRKCYARLHPRAVNCRKKKCGHSNQLRPKKKIK

>Glyma15g41230.1

MQIFVKTLTGKTITLEVESSDTIDNVKAKIQDKEGIPPDQQRLIFAGKQLEDGRTLADYNIQKESTLHLVLRL
RGGTMIKVKTLTGKEIEIDIEPTDTIDRIKERVEEKEGIPPVQQRLIYAGKQLADDKTAKEYNIEGGSVLHLV
LALRGGTY

>Glyma13g17820.1

MQIFVKTLTGKTITLEVESSDTIDNVKAKIQDKEGIPPDQQRLIFAGKQLEDGRTLADYNIQKESTLHLVLRL
RGGMQIFVKTLTGKTITLEVESSDTIDNVKAKIQDKEGIPPDQQRLIFAGKQLEDGRTLADYNIQKESTLHLV
LRLRGGMQIFVKTLTGKTITLEVESSDTIDNVKAKIQDKEGIPPDQQRLIFAGKQLEDGRTLADYNIQKESTL
HLVLRLRGGMQIFVKTLTGKTITLEVESSDTIDNVKAKIQDKEGIPPDQQRLIFAGKQLEDGRTLADYNIQK
ESTLHLVLRLRGGMQIFVKTLTGKTVTLEVESSDTIDNVKAKIQDKEGIPPDQQRLIFAGKQLEDGRTLADY
NIQKESTLHLVLRLRGGF

>Glyma09g02760.1

MQIFVKTLTGKTITLEVESSDTIDNVKAKIQDKEGIPPDQQRLIFAGKQLEDGRTLADYNIQKESTLHLVLRL
RGGAKKRKKKTYTKPKKIKHKHKKVKLALLQFYKVDDSGKLQRLRKECPNAECGAGTFMANHFDRHYC
GKCGLTYVYQKSEA

>Glyma08g17870.1

MQIFVKTLTGKTITLEVESSDTIDNVKAKIQDKEGIPPDQQRLIFAGKQLEDGRTLADYNIQKESTLHLVLRL
RGGTMIKVKTLTGKEIEIDIEPTDTIDRIKERVEEKEGIPPVQQRLIYAGKQLADDKTAKEYNIEGGSVLHLV
LALRGGTY

>Glyma06g13870.1

MQIFVKTLTGKTITLEVESSDTIDNVKAKIQDKEGIPPDQQRLIFAGKQLEDGRTLADYNIQKESTLHLVLRL
RGGIIEPSLMALARKYNQDKMICRKCYARLHPRAVNCRKKKCGHSNQLRPKKKIK

>Glyma04g40980.1

MQIFVKTLTGKTITLEVESSDTIDNVKAKIQDKEGIPPDQQRLIFAGKQLEDGRTLADYNIQKESTLHLVLRL
RGGIIEPSLMALARKYNQDKMICRKCYARLHPRAVNCRKKKCGHSNQLRPKKKIK

>Glyma03g35540.1

MQIFVKTLTGKTITLEVESSDTIDNVKAKIQDKEGIPPDQQRLIFAGKQLEDGRTLADYNIQKESTLHLVLRL
RGGIIEPSLMALARKYNQDKMICRKCYARLHPRAVNCRKKKCGHSNQLRPKKKIK

>Glyma02g04090.1

MQIFVKTLTGKTITLEVESSDTIDNVKAKIQDKEGIPPDQQRLIFAGKQLEDGRTLADYNIQKESTLHLVLRL
RGGAKKRKKKTYTKPKKIKHKHKKVKLGILQFYKVDDSGKVQRLRKECPNAECGAGTFMANHFDRHYC
GKCGLTYVYQKADA

>Glyma01g03570.1

MQIFVKTLTGKTITLEVESSDTIDNVKAKIQDKEGIPPDQQRLIFAGKQLEDGRTLADYNIQKESTLHLVLRL
RGGAKKRKKKTYTKPKKIKHKHKKVKLGILQFYKVDDSGKVQRLRKECPNAECGAGTFMANHFDRHYC
GKCGLTYVYQKADA

>Glyma17g04700.1

MQIFVKTLTGKTITLEVESSDSIENVKAKIEEKEGIPPDQQRLIFAGKQLEDGRTLEDYEIQKESTLHLVLRLR
GGMQIFVKTLTGKTITLEVEGSDTIENVKAKIQEKEGIPPDQQRLIFAGKQLEDERTLEDYDIQKESTLHLVL
RLRX

>Glyma19g23760.1

MQIFVKTLTGKTITLEVESSDTIDNVKAKIQDKEGIPPDQQRLIFAGRQLEDGRTLADYNIQKESTMHLPLRL
HX

>Glyma18g25870.1

MQIFVKTLTGKTITFKVESRDTIDNVKGKIQDKEDQQCLIFVEKQLEDGRTLADYNIKKESTFHLLLKLRGGI
IKPSFMALARKYNLDKMIFHN

>Glyma07g32040.1

IFVKTLIGKTITLEVESSDTIDNVKAKIQDKESIPPDQQRLIFAGKQLEDGLTLADYNIQSLPFTLSFV

>Glyma15g35160.1

FVKTLTGKTITLEVESSDTIDNVKAKIQDKHSSGSTATLADYNIQKESTLHLVLRLRGGIIEPSLMALARKYN
QDKMIFHX

>Glyma09g16000.1

MDQQRLIFVGKQLEDGRTLVDYNIQNESTLHLVLRLRGGMQIFVKTLIGKTITLEVESCDTIDNVKAKIQDK
EAIGRWSYLGRLRYSKGIHLASSTSPSWWLLSDVSLERVLCSSSLSVVVYYSFQTILSLTLQDHQSTKEFSSS
ANCK

>Glyma14g39850.1

MAGQSSNEGSSTGNISAECSDSTVQLNIKTLDSRIYSFQVDKNMPVSLFKEKIANEIGVPVNQQRLIFRGKVL
KDEHVLSEYHVENGHTLHLVERQPNQSQASGMSSGESTGTSGNRGNGVGSGAPRNRVGQISHSVVLGTFN
VGEQGEGIVHDLTRVIGHVLNSIGNSGQSTISGPNATQTSSVQPRNETDGMHAGNQNPAGNQAPSGQTFHG
PTFQSVSHVVQIPVAAGTIPIPSLNAPIPDSLNTLSEFMNRMEQILTQNGYQSNLSSANPRDQLVELPSNVQG
LPTLEALSTVLHRAEQLLGGQAVAALSHIAGRLEREGTSADLHIRGQIQSESVQIGLAMQHLGALLLELGRT
MLTLRMGQSSAESVVNAGPAVYISPSGPNPIMVQPFPLQTSSLFGGPVPPSTPATLGTIGIGNAPRNVNIHIHA
GTSLAPIVSAIGSRENNGEGTRSEHHNEPGSGDSGSTRVLPVRNVIAATIPSHPPGVGVSSSTQTGFGISTSQP
PSDSASLSSVLAEINSRLRNVVGNMQGDNTVPSGQMESNSRDLSSGSESRPPTVNKQQDTVDVNGFGAISA
SSVGCTSESEVQKVQTEAVQTSSNVLVDKFVSSSSNQDLQSCSSGETIVKPEIEQDVLAVSERQNVTEPAKA
APLGLGVGGLERKRRTRLQPPVSKGADDGSSSSSVNQNQQTRTDGQHILQTLASHGSGLNSRNANGPSQRP
LPSSDRPIDVAGLMSQALHSPALNGLLEGVSQQTGVDSPDGLRNMLHQFTQSPQMMNTVNQIVQQVGSQD
VGNMFAGTERGQGGGIDISRMFQQMMPIVSRALGGGNPSSLFSAEEAEPHAPYRDGTVDRDEYSDNQSLQ
LYLQPLAERIEHLGPSTDIFRAVAEIAVQLSGSGSTSNDLLDELCSNESLAREYVDMLRFDVSKLLEGHSETD
NV

>Glyma07g39550.1

MRKDEAKAPGAMPQQQRSHIQFFVRMMSAGNTIVMQAFPEDTGIPLFEQRLIYRGKQLQWEQTLAECFIQ
NDANLQLVGRMRSTEHPQAWQVINDMVSLVYRLCRGETVHDALKTVKGLMTSYLNMTPRIDNDSASGYF
QIFMSSSAPAVLVMLYVSPYAGNKDCADSSVRHFLSSCRNILSKALHGQCARVVLEFCKLLRRVGSHDPLY
LFCRSTFGSLLETAGVSYGSGSDNVKGLVLIQDIFPFVCELANSLLRDLDLSIVSPSAAGPLSNDVGDFSAFL
LPLRTGIKEQQAVKDSMAQDKHHKLAEEIEYLHGLYVQLLNKIDQCLQKMDQSLAGQEMMEGDNLYPA
WSHYLSILKELYQISKLYDGAEEKLWGVLTRQRSVLCLLIVRYAKRTDEHQWILEHRYVTNFESRRHLAM
MMFPEVKEDYEDLLNTSHVLSLTPCMLACLWNSKTRKLLAQAKAVNSWSASSSSSSSDAGGCSSLQQQQR
SHIQFFVRMMSAGNTIVMQAFPEDTVKSIHERIQSMKGIPLFEQRLIYRGKQLQWEQTLAECFIQNDANLQL
VGRMRSTEHPQAWQVINDMVSLVYRLCRGETVHDALKTVKGLMTSYLNMTPRIDNDSASGYFQIFMSSSA

PAVLVMLYVSPYAGNKDCADSSVRHFLSSCRNILSKALHGQCARVVLEFCKLLRRVGSHDPLYLFCRSTFG
SLLETAGVSYGSGSDNVKGLVLIQDIFPFVCELANSLLRDLDLSIVSPSAAGPLSNDVGDFSAFLLPLRTGIKE
QQAVKDSMAQDKHHKLTEEIEYLHGLYVQLLNKIDQCLQKMDQSLAGQEMMEGDNLYPAWSHYLSILKE
LYQISKLYDGAEEKLWGVLTRQRSVLCLLIVRYAKRTDEHQWILEHRYVTNFESRRHLAMMMFPEVKEDY
EELHEMLIDRSQLLTESFEYIARAEPDSLHAGLFMEFKNEEATGPGVLREWFLLVCQAIFNPQNALFVACPN
DRRRFFPNPASKVHPLHLEYFSFAGRVIALALMHRVQVGIVFDRVFFLQLAGNYIAIEDIRDADPYLYTSCK
QILDMDADFIDSDSLGLTFVREVEELGQRKVVELCPGGKNLVVNSKNRDKYVDLLIQDRFVTSISEQVSHF
VKGFADILSNSKLQQYFFQSLDLEDLDWMLHGSEDTISVEDWKAHTEYNGYKETDIQISWFWEIVGRMTA
DQRKVLLFFWTSVKYLPVEGFRGLASRLYIYRSLEPGDRLPSSHTCFFRLCFPAYSSMAVMKDRLEVITQEH
IGCSFGTW

>Glyma17g38130.1

MAGQSSNEGSSTGNISAECSDSTVQLNIKTLDSRIYSFQVDKNMPVSLFKEKIANEIGVPVNQQRLIFRGKVL
KDEHALSEYYVENGHTLHLVERQPNQSQASGTSSGNDVGSGAPRNHVGQISHSVVLGTFNVGEQGEGIVH
DLTRVIGHVLNSIGNGGQSTLSGPNATQTSSVHPWNETEGMHAGNQNSAGNQAPSGQTFHGPTVQSVSHV
VQIPVAAGAIPIPSLNAPIPDSLNTLSEFMNRMEQTLTQNGYQSNLSSANPGDQQAELPSNAQGLPTLEALST
VLHSAERLLGGQAVAALSHIAGRLEREGTSADLRVRDQIQSESVQIGLAMQHLGALLLELGRTMLTLRMG
QSSAESVVNTGPAVYISPSGPNPIMVQPFPLQTSSLFGGPVPPSTPATLGTIGIGNAPRNVNIHIHAGTSLAPIV
SAIGSGANNGEGTRSEHRNEPGSGDSGSTRVLPVRNVIAATIPSHPPGVGISSSTQTGFGIPTSQPPSDSASLSS
VLAEINSRLRNVVGNMHGDNTVPSGQMESNSRDLPSGSESRPATVNEQRDTMDMNGFGATSASSVGCTSE
SEVQKLQTKAVQTSSNDERDVLVDKFVSSSSNQDLRSCSSGETIVKPEKEQDVPAVSERQNVTEPAKAAPL
GLGVGGLERKRRTRLQPPVSKGADDRSSSSSANQNQQTRTDGQHILQTLASHGSGLNSRNANGPSQRSLPS
SDRPIDVAGLMSQALRSPALNGLLEGVSQQTGVDSPDGLRNMLQQFTQSPQMMNTVNQIVQQVGSQDVG
NMFAGMERGQGGGIDISRMFQQMMPIVSQALGGGNPSSLFSAEEAEPHAPYCDGTINRDEYSDNQSLQLDL
QPLAERIEHLGPSTDIFRAVAENAVQLSGSGSTSNDLLDELCSNESLAREYVDMLRCDVSKLLKEHSETNNV

>Glyma08g32090.1

MKVFVKTLKGTHFEIEVNPSDTLSEVKKNIETVQGADVYPAAQQMLIHQGKVLKDGTTLEENKVAESSFIV
IMLSKTKSSSGEGSTTSTAPSAKPSATPTSTSVSTAPQAPASTGASATPVTAPTAAPAPAPAPAAPAPAPAPIF
SGSSVRPESDIYGQAASNLVAGSNLEGTIQQILDMGGGSWDRDTVVRTLRAAYNNPERAVEYLYSGIPEQA
EAPPVTGVPASAQPSNPPADTPQAAQPASVPSSGPNANPLDLFPQGLPNVGSGAAGAGSLDFLRNSQQFQA
LRAMVQANPQILQPMLQELGKQNPHLMRLIQEHQVDFLRLINEPVEGGEGNILGQLAGAMPQAVTVTPEE
RQAIERLEAMGFDRATVLEVYFACNKNEELAANYLLDHMHEFDE

>Glyma01g20670.1

MKVFVKTLKGTHFEIEVNPSDTLSEVKKNIETVQGADVYPAAQQMLIHQGKVLKDGTTLEENKVAENSFIV
IMLSKTKSSSGEGSTTSTAPSVKASATPTSTSVSAAPQAPASTGATPTPVTAPAAPASAAAPAPISSGSAVPES
DIYGQAASNLVAGSNLEGTIQQILDMGGGSWDRDTVVRALRAAYNNPERAVEYLYSGIPEQAEAPPVTREP
ASAQPANPPAAAPQAAQPASVPSSGPNANPLDLFPQGLPNVGSGAAGAGSLDFLRNSQQFQALRAMVQAN
PQILQPMLQELGKQNPHLMRLIQEHQVDFLRLINEPVEGGEGNILGQLASAMPQAVTVTPEERQAIERLEA
MGFDRATVLEVYFACNKNEELAANYLLDHMHEFDE

>Glyma17g11440.1

MGSTEKIPISAESSETTIEIKIKTLDSQTYTLRVDKQMPVPALKEQIASVTGVLSERQRLICQGKVLKDDQLLS
AYHVEDGHTLHLVVRQPDLPPPGSVSNHSVTELNSSTGLGHASQVAPGVFIETFNVPVQGDGVAPEINRIVS
AILGSIGLPNFASGSEGGIDVREHDSQGSGRTLGSSGILDSSHPRPEQAGLRILSDRLRNTFGTPAPVSLGSLQ

PPVIPDSLTTLSQYLRHISLEFDAIVREGRDNAEAAEAQRNEETRSVSSHLGSTPEGLSSPASLAEVLRSTRQ
MIDQQAGECLLQLSGQLENQANVTDPLLRSSIQSRALRTGVLFYNLGAFLLELGRTTMTLRLGQTSSEAVV
NGGPAVFISPNGPNHIMVQPLPFQPGASFGAVPVGAAQSNSSLGSGLGSSFFPRRIDIQIRRGTSTTSSNTNQE
ERNETQSASVQRNSGESSVNQATSRRPDASIAGEPGVRLVPIRTMVAAAVPGTLGRLPSESSGNSIGLYYPIL
GRFQHVSSGHSNSEQGSQQSSQHHTVLPSTPESILQRQNTEDSARNVGSLSTPSTRQEPSSSRVVNINILAAS
GPQNNQESERQIPSSVLQFLRTFFPGGEIHVEDSSVQGTTAGSALDHAATSRGAAPVPEAQPNVSEEGIFLSN
ILREIMPVISQQVGSEGNPSEDHMAQDSSTQVETDVGTSRRQSDSDPSPPNAKRQKME

>Glyma04g14730.1

DEEEYEESGKGNRFLGFMFGNVDNSGDLDVDYLDEDAKEHLSALADKLGPSLTDIDLSGKSPQTPPDVVE
QGCDVKAEDAVDYEDIDEEYDGPETEAANEEDYLLPKKEFFSAEASVCLESKASVFDDENYDEDSEKEQD
FLENLWKEKNDWFSLINGEQKESFVDASKEESSLEHELHVDSPQTEELDADVQKLEEESPEVPKRSMAMPL
PVLCVEDGVTILRFSEIFGIHEPLRKGEKREHRHSIPRDRYKSLDLIDDFIEEDEEEFLKGFSQSLSLTKQVCV
VHNDVSESNDVDLEFPKFGFLLADASDSCHSAEPMKGDFAEDHSRKDHPFMLANFYPLDQQDWEDEILW
GNSPVPSNNNVESCEISGPELGASGGSEIEIESGIQSIQMEPQKKLEDKDHNVLMCSSPVKVEPFGSWDSFGA
KTNLISRSLFHPQLLRLESRSEVDSSSLADGREAEISEHNQSGQVKRFTKVISQNRDMMEGSWLDKIIWEEL
DQPMVKPKLIFDLQDDQMHFEVLDSKDGTHLRLHAGAMILTRSLQSISGDSSELPGHGSQYGWRHVANDK
HYSNRKTSQQLKSNSKKRSAHGVKVFHSQPALKLQTMKLKLSNKDIANFHRPKALWYPHDNEVAVKEQG
KLPTQGPMKIIIKSLGGKGSKLHVDAEETLSSVKAKASKKLDFKVSETVKIFYLGRELEDHKSLAAQNVQPN
SLLHLVRTKIHLWPKAQRVPGENKSLRPPGAFKKKSDLSVKDGHVFLMEHCEERPLLLSNVGMGARLCTY
YQKCSPDDQSGSLLRNTDNSLGHIISLDPADKSPFLGDLKPGCTQSSLETNMYRAPVFPHKVPLTDYLLVRS
SKGKLSLRRIDKINVVGQQEPLMEVLSPGSKNLQNYMINRLLVHMCREFQAAEKRHMPPYIRVDEFLSQFP
YQSEASFRKKIKEYANLQRGTNGQSILVKKRNFRIWSEDELRKMVTPELVCAYESMQAGLYRLKHLGITET
HPTNISSAMSRLPDEAIALAAASHIERELQITPWNLSSNFVACTSQGKENIERMEITGVGDPSGRGMGFSYAR
APPKAPVSSAMVKKKAAANRGGSTVTGTDADLRRLSMDAAREVLLKFNVPDEVIAKQTRWHRIAMIRKL
SSEQATSGVKVDPTTISKYARGQRMSFLQLQQQTREKCQEIWDRQVQSLSAVNGDENESDLEGNSDLDSFA
GDLENLLDAEECEEGEESTNDLKRDKGDGVKGLKMRRHPTLAQAEEEIEDDAAEAAELCRLLM

>Glyma09g34820.1

MDDEEEYEDSGKGNRFLGFMFGNVDNSGDLDVDYLDEDAKEHLSALADKLGPSLTDIDLSGKSPQTPPDV
VEQDCDVKAEDAVDYEDIDEEYDGPETEAANEEDYLLPKKEFFSSEASVCLESKASVFDDENYDEESEKEQ
DFDGPEVQKRSMAMPLPVLCVEDGVAILRFSEIFGIHEPLRKGEKREHRHSIPRDIYKSFDLTDDFVEEDEEE
FLKGFSQSLSLSKQVCVVHNDVSESNDVDLEFPKFGFLHADASVDRKDDQQSKDSCHSAEPMKGDFVEDH
FWKDHPFMLANFYPLDQQDWEDKILWGNSPVPSYNNVESCEISGPELGASGGSEIEIESGIHNIQMEPQKVL
EDKNHNVLMRSSPVKLEPFGSRDSSGAKTNLISRSLFHPQLLRLESRSEVDSSSLADGRDAEISEHNQSGQV
KRFTKVISQNRDMMEGSWLDKIIWEELDQPSVKPKLIFDLQDDQMHFEVLDTKDGTHLCLHAGAMILTHS
LKLSSGDSSELPGHGSQYGWRYVANDKHYSNRKTSQQLKSNSKKRSAHGVKVFHSQPALKLQTMKLKLS
NKDIANFHRPKALWYPHDNEVAVKEQGKLPTQGPMKIIIKSLGGKGSKLHVDVEETLSSVKAKASKKLDF
KVSETVKIFYLGRELEDHKSLAAQNVQPNSLLHLVRTKIHLWPKAQRVPGENKSLRPPGAFKKKSDLSVKD
GHVFLMEYCEERPLLLSNVGMGARLCTYYQKCSPDDQSGSLLRNTDSRLGHIISLDPADKFPFLGDLKPGC
SQSSLETNMYRAPIFPHKVPLTDYLLVRSSKGKLSLRRIDKINVVGQQEPLMEVLSPGSKNLQTYMMNRLL
VHMCREFQAAEKRHLPPYIGVDEFLSQFPYQSEASFRKKIKEYANLQRGTNGQSILVKKRNFRIWSEDELRK
MVTPELVCAYESMQASLYRLKHLGITETHPTNISSAMSRLPDEAIALAAASHIERELQITPWNLSCNFVACTS
QGKENIERMEITGVGDPSGRGMGFSYARAPPKAPVSSAMVKKKAAANRGGSTVTGTDADLRRLSMDAAR
EVLLKFNVPEEVIAKQTRWHRIAMIRKLSSEQATSGVKVDPTTISKYARGQRMSFLQLQQQTREKCQEIWD
RQVQSLSAVNGDENESDSEGNSDLDSFAGDLENLLDAEECEEGEEGTNDLKRDKGDGVKGLKMRRRPTL
AQAEEEIEDEAAEAAELCRLLMDDYEADRKKKKKAKVMVGEARLVPKMQSKFSFDNAEQEENVPAKKSK
SLKVNKAKKNDIMPISIPNKKIKLNMGEGIKNQVFKEKKPSRETFVCGACGKAGHMRTNKNCPKYGEDLE

TQLESADMEKSSGKSSFVDPSSLSQHKAPSKNSTEKSSDKPAVETLQSSDKPVTSDSETAKSAKVNKIIIPKK
VKPDDTLAESRKHAIVIRPPTDSGRGQVDSHKFPIKIRPPTEIDREQSHKKIVIKRTKEVIDLELDSPGGNTGL
QHRKTKRIVELSNFEKQKKQETVYGTEGFKKWNSKEDRRWREEQEKWRNDARLREEDRARRHHKEEIRM
LKEQERLDEIKRFEEDIRREREEEERQKAKKKKKKKPELRDEYLDDPRARRHDKRMPERDRSGKRRSVTE
LGKIGADYMPPTKRRRGGGGEVGLANILESVVDTIVKDRYDLSYLFLKPVSKKEAPDYLDVIERPMDLSRIR
ERVRNMEYKSREDFRHDMWQITFNAHKYNDGRNPGIPPLADMLLEYCDYLLNENDDSLTEAEAGIEIRDF

>Glyma13g44910.1

MKLTVKTLKGSHFEIRVQPSDTIMAVKKNIEDVQGKDNYPCGQQLLIHNGKVLKDETTLSDNKVSEDGFL
VVMLSKGKTLGSAGISSTQFASNPPTTVSTPNSTPLVQPQSANNNASATDVTTTNVTTNTYGQAASNLVAG
SNLEQTIQQLMDMGGGSWDRDTVTCALRAAYNNPERAVDYLYSGIPVAAEIAVPAASYPISQTTETGGAS
VGAVPGVPNSSPLNMFPQETISGAGAEIGSLDFLRNNPQFQALRSMVQSNPQILQPVLQELGKQNPSLLTLIQ
EHHAEFLQLINEPVEGSEGDIFDQPEQDMPHAINVTPAEQEAIGRLEAMGFDRASVIEAFLACDHDEQLAAN
YLLENAGDFEG

>Glyma04g41450.1

MACVALHPVCEESLNRGEFTSCFSPHLRESILIYLTVGGSVIPMHIMKTDSIASVKLRIQTFKGFFVKKQKLV
FEGKELARNRSCIRDYGVGDGNVLHLVLRLSDLKAITVRTMCGKEFGLYVEKSRSVGYVKQQIAKKGQGF
LDHKDQELICEGEELEDQRLIEDICKDNDAVIHFLVRKSDAKVRTKPVDKDFELSIEASYVHNLRSQLTRDF
VLEPIIMNSNIKIPLVIQELIKSTSEGLEKGCKPIQSSEGSGGAYLMQDSSGLKYVSVFKPIDEEPMAINNPRGL
PVSEDGEGLKKGTRVGQGALREVAAYILDHPRKGPRSYYNNEEGGFAGVPPTVMVKCLDKGFHNIEDYQ
NDSANVKIGSLQMFMRNIGSCEDMGPSAFPVEEVHKISVLDIRLVNADRHAGNILIARDGEDGQTILIPIDHG
YCLPESFEDCTFDWLYWPQAKEPYSPDTIDYIKSLDAEEDIKLLKFHGWDLPPKCAQILRISTMLLLKGAER
GLTPFALGSIMCRETLKKKSVIEQIVEEAEEAAFPGASEAAFLDLVSVVMDSHLDELFP

>Glyma08g02730.1

EPILIYLTVDGAVTPMRVLESDSIASVKLRIQQCKGFVVKKQKLVFSGRELARNGTLIKEYGVTDGNVLHLV
LRLSDLLFIVVRTVSGKEFEFHIDRHRNVGYLKQRIRKNKGECFIDLEDDDQEFFCNDQKLDDDSLFHDICK
SGDDVIHLIIKKSVKVRTTPIHKDLNLSVVAPGESVKHKRDKHVQIAKVPPDVGFWLEPIIVNPKIIFFPFLWD
MVKSTFEGLKKGNHPVRSSEGTGGTYLMQDSTGQEHVSVFKPMDEEPMAVNNPKGLPNSSNGEGLKRGT
KVGEGAFREVAAYLLDHPKSGPRLASGEAVGFSGVPPTVMVQCLHQEFNHPNGFASSSKHVKIGSLQKFIS
NDGNCEDYGPSAFPVEEVHKIAVLDIRLANADRHAGNILIKKEADGQIKLIPIDHGYCLPDKFEDCTFDWLY
WPQVRQPYSPETVNYINSLDAEKDLELLKCYGWDIPLECARTLRISTMLLKKGVERGLTPYAIGSIMCRENL
NKESVIEEIIGEAQDSLLPGMEESAFLEAISQIMDYHLDKLAN

>Glyma20g24850.1

MKVFVKTLKGTHFEIEVTPQDTVSEVKKNIETVQGADVYPAAQQMLIHQGKVLRDATTLEENKVAENTFI
VIMLSKSKSTSGEGSTTSTALSTKAPQTSTVPASTPPVSVAPQAPAPAATGALPASVTAPVSSPSPAPAPAPA
PISSGTAVEGSDIYGQAASNLVAGSNLEGTIQQILDMGGGSWDRDTVVRALRAAYNNPERAVEYLYTGIPE
QAEAPLVARAPVSAQPTNPPADAPQTAQPAAVTSAGPNANPLDLFPQGLPNVGSGAAGAGSLDFLRNSQQ
FQALRAMVQANPQILQPMLQELGKQNPHLMRLIRDHQADFLRLINEPAEGGEGNILGQMASGMPQAVTVT
PEERQAIERLEAMGFDRAIVLEVYFACNKNEELAANYLLDHMHEFEEQ

>Glyma18g08070.1

MKITVMTADEQIITLDVDPHESVENVKALLEVETSVPLQQQQLLFNGKEVRNSEKLSALGVKDDDLLMMV
SGAGAGATASSGSTNDLSLNADGSAVNPGSFQQHIRHDSNLMSQLFQSDPELAQAILGNDLNKLQEVLRLR

HHQRDELKRQKEEELALLYADPFDVEAQKKIEAAIRQKGIDENWAAALEHNPEAFARVVMLYVDMEVNG
VPLKAFVDSGAQSTIISKSCAERLGLLRLLDQRYRGIAHGVGQSEILGRIHVAPIKIGSIFYPCSFLVLDSPNM
EFLFGLDMLRKHQCIIDLKENVLRVGGGEVSVPFLQEKDIPSRFLDEEKYAKEASGSGGQTKVSSSILTFFSC
NQKERCLISRILIAIIIFLFLFPYGFKSKRQFLSVKLKLCSDYCQFMILSLKPKLQSLLNWDLKEMQLYKLFNY
SMAMRNRQLGFFLGARNNPIQLQILNHRHDGIYCVSCSYFKECFYSIEKTNSMFLFI

>Glyma10g42180.1

MKVFVKTLKGTHFEIEVTPQDTVSEVKKNIETVQGADVYPAAQQMLIHQGKVLRDASTLEENKVVENTFI
VIMLSKSKSPSGEGSTTSTAPSTKAPQTSTVPASTPPASVVPQASAPAPAPAPAATGALPASVTAPISSPSPAP
APTPAPISSGTAVEGSDIYGQAASNLVAGSNLEGTIQQILDMGGGSWDRDTVVRALRAAYNNPERAVEYLY
TGIPEQAEAPLVAQVPASAQPTNPPADAPQTAQPAPVTSAGPNANPLDLFPQGLPNVGSGAAGAGSLDFLR
NSQQFQALRAMVQANPQILQPMLQELGKQNPHLMRLIRDHQADFLRLINEPAEGAEGNILGQMASAMPQA
VTVTPEERQAIERLEAMGFDRAIVLEVYFACNKNEELAANYLLDHMHEFEEQ

>Glyma08g23930.1

MKLTVKTLKGSHFEIRVQPSDTVMAVKKNIEDVQGKDNYPCGQQLLIHNGKVLKDETTLVENKVSEDGFL
VVMLSKSKTSGSAAASSVQPASNPPTTVSTSNSTPPSDPPVQTQAANNSTSSTDAPTTNVSADTYGLAASNL
VAGSNLEQTIQQIMDMGGGNWDRDTVSRALRAAYNNPERAIDYLYSGIPEAAEVAVPVPQTAGISSGAVP
VGPNSSPLNMFPQETISSTGAGLGSLDFLRNNPQFQALRSMVQSNPQILQPVLQELGKQNPGLLRLIQEHHG
EFLQLINEPVDGSEGMKGTFLLVSSICYSYNIIDIFEQPEQDMPHAINVTPAEQEAIGRLEAMGFDRASVIEAF
LACDRDEQLAANYLLENAGDFED

>Glyma07g00490.1

MKLTVKTLKGSHFEIRVQPSDTVMAVKKNIEDVQGKDNYPCGQQLLIHNGKVLKDETTLVENKVSEDGFL
VVMLSKSKTSGSAAASSVQPASNPATTVSMSNSTPPSDPPVQTQAANNSTSSTDAPTTNVSADTYGLAASN
LVAGSNLEQTIQQIMDMGGGNWDRDTVSRALRAAYNNPERAIDYLYSGIPEAAEVAVPVPQTAGMSSGAV
PVGPNSSPLNMFPQETISSTGAGLGSLDFLRNNPQFQALRSMVQSNPQILQPVLQELGKQNPGLLRLIQEHH
GEFLQLINEPVEGSEGDMFEQPEQDMPHAINVTPAEQEAIGRLEAMGFDRASVIEAFLACDRDEQLAANYL
LENAGDFED

>Glyma16g22530.1

IISSLSVIRVSSRQLAESLDPGFLTLGLGVRVTRLPRDAMGGDSAIEESEGVNVNINVRCSNGFKFSVQIAVDS
TVSSFKDVVARNCDIPAEQQRLIYKGRILKDDQTLRSYGLEADHTVHLVRGSAPANPTGGTNTSSTNTNTT
TNNARGAGANEGGGLGGLGFGASLFPGLGVNGTGGNGLFGEGFPDLEQMQQPFISNPNLVREIMNSPTMQ
NLMNNPEIVRNLIMNNPQMQELMDRNPELAHILNDPSTLRQTLEATRNPEIMREMMRNTDRAMSNIESSPE
GFNMLRRMYENIQEPFLNATTMAGNTGNNNAAVSGTHGGHARDRSTNPSTTSSEATAGSPLPNTNPLPNP
WSSTGTGGAQNNPRRSLTTGVDARQQGPTGLGGLGLPDLESMMGGSAMPDAALLTQLMQNPAISQMMQ
SMLSNPQTLNQILGANTEQRGMPDLNSLREVMQNPEFLRLFSSPETLQQLLSFQQALMSQLGQQQSTRESG
QTGGGTGPMNNLGLEMLSSMFGGLGAGSLAVPNRSNEPPEQLYASQLSQLQEMGFFDTQENIRALIATSGN
VHAAVERLLGNSGQ

>Glyma03g41850.1

MSSAGVSTLTVVPTEPLLSPKAFPFPIPSHLSLEDKSIFIYLSFSGSLTPIRVMEWDTIESVKFKIQRCESLPFLT
NKQKLVYAGRELARSDTPLKDYGVTDGNVLHLVIKLSDLQVINVKTSCGKEFTFQVERGRDVGYIKQRIAR
REKQFDDPEEQELVCNGERLEDQRLIDDICCKHNDAAVHLFVRKKHVKVQRRPLELSIVAKDLIDKKKNDP
PGRDFILEPVIINHKIELAPAIRNMVNSTYEGLGSGKCPIRSAEGTGGAYFMLDSAEQKYVSVFKPIDEEPMA

VNNPRGLPLSLDGEGLKKGTRVGQGAFREVAAYVLDHPLSGCQRHSLFGDGKGFAGVPPTLMVKCLHKA
FNYPRELTPKIGSLQMFTENSGSCEDMGPGAFPVKEVHKITVLDIRLANADRHAGNILISKEEDNNQSVLIPI
DHGYCLPTSFEDCTFEWLYWPQARQPYSSETIDYIKSLDAEEDIALLKFHGWDLPVECARTLRISTMLLKKG
VERGLTPFAIGSLMCRESLNKESVIEEVVQAALDSVLPGTSEATLLDSVSQILDLHLDEIVRSHL

>Glyma19g44510.1

MSSAGVSTLSVVPTEPLLSPMAFPFPIPSHLSLEDKSIFIYLSFSGSLTPIRVMEWDTIESVKFKIQRSESLPFLT
NKQKLVYAGRELARSDSLLKDYGVTDGNVLHLIIKLSDLQVISVKTSCGKEFTFQVERGRDVGYIKRRIAR
REKQFDDPEEQELVCNGERLEDQRLIDEICCKHNDAVVHLFVRKKHVKVQRRPLELSIVAKDLINKKKNDP
PDRDFILEPVIINPKIELAPAIWNMVNSTYDGLGSGKYPIRSAEGTGGAYFMIDSAGQKPMALNNPRGLPLSL
DGEGLKKGTRVGQGAFREVAAYVLDHPLSGRRSLFGDGKGFAGVPPTLMVKCLHKSFNYPREFTPKIGSL
QMFTENSGSCEDMGPGAFPVKEVHKITVLDIRLANADRHAGNILISKEEDNNQSVLIPIDHGYCLPTSFEDCT
FEWLYWPQARQPYSPETIDYIKSLDAEEDIALLKFHGWDLPVECARTLRISTMLLKKGVERGLTPFAIGNLM
CRESLNKESVIEEIVQAALDSVLPGTSEATLLDAVSQIMDLRLDEIVRARL

>Glyma02g04740.1

MGGDSAVEESEGVNVNINVRCSNGSKFSVQIAVDSTVSSFKDAAACSCDIPAEQQRLIYKGRILKDDQTLRS
YGLEADHTVHLVRGSALANPTGGTNTSSTDTNTNTNIPTDNARVAGANEGEGLGGLGFGASLFPGLGVNG
TGGNGLFGEGFPDLEQMQQPFISNPNLVREIMNSPTMQNLINNPEIVRNLIMNNPQMQELMDRNPELAHILN
DPSTLRQTLEATRNPEIMREMMRNTDRAMSNIESSPEGFNMLRRMYENIQEPFLNATTMAGNTGNNNAAV
SGTHGGHARDPSTNPSTTSSEATAGSPLPNTNPLPNPWSSTGTGGAQNNSRRSLTTGVDARQQGPTGLGGH
GLPDLESMLGGSAMPDPALLTQLMQNPAISQMMQSMLSNPQTLNQILGANTEQRGMPDLNSLREVMQNP
EFLRLFSSPETLQQLLSFQQTLMSQLGQQQSTWESGQTGGGTGPMNNLGLEMLSSMFGGLGAGSLAVPNR
SNEPPEQLYASQLSQLQEMGFFDSQENIRALIATSGNVHAAVERLLGNSGQ
