# Supplementary material for: High level transgenic expression of soybean (Glycine max) GmERF and Gmubi gene promoters isolated by a novel promoter analysis pipeline
Source: BMC Plant Biol. 2010 Nov 4;10:237. doi: 10.1186/1471-2229-10-237 (PMC3095320; doi:10.1186/1471-2229-10-237)
Supplement: Additional file 2 — List of AP2 domains used for the phylogenetic analysis of the GmERF genes. The soybean GmERF genes were obtained from SoyDB: A Knowledge Database of Soybean Transcription Factors (http://casp.rnet.missouri.edu/soydb/) and verified using the Soybean Transcription Factor Knowledge Base (http://www.igece.org/Soybean_TF/). [file 1471-2229-10-237-S2.PDF]

Additional file 2. List of AP2 domains used for the phylogenetic analysis of the *GmERF* genes. The soybean *GmERF* genes were obtained from SoyDB: A Knowledge Database of Soybean Transcription Factors (http://casp.rnet.missouri.edu/soydb/) and verified using the Soybean Transcription Factor Knowledge Base (http://www.igece.org/Soybean_TF/).

> Glyma20g16920.1 (GmERF1)

KRYRGVRRRPWGKFAAEIRDPTRKGTRVWLGTFDSEIDAAKAYDCAAFKMRGQKAILNFPLEA

> Glyma20g16910.1 (GmERF2)

KHYRGVRRRPWGKYAAEIRDPNRKGSRVWLGTFDTAIEAAKAYDKAAFKMRGSKAILNFPLEI

> Glyma11g03900.1 (GmERF3)

KHYRGVRQRPWGKFAAEIRDPNKRGSRVWLGTFDTAIEAAKAYDRAAFRLRGSKAILNFPLEA

> Glyma01g41530.1 (GmERF4)

EKKHYSATETLGKFAAEIRDPNKRGSRVWLGTFDTAIEAAKAYDRAAFRLRGSKAILNFPLEA

> Glyma05g05180.1 (GmERF5)

KHYRGVRQRPWGKFAAEIRDPAKNGARVWLGTFETAEDAALAYDRAAYRMRGSRALLNFPLRV

> Glyma05g05130.1 (GmERF6)

KHYRGVRQRPWGKFAAEIRDPNKRGSRVWLGTFDTAIEAAKAYDRAAFRLRGSKAILNFPLEV

> Glyma19g43820.1 (GmERF7)

VSYRGVRRRPWGKFAAEIRDSTRNGVRVWLGTFDTAEAAALAYDQAALVMRGSMAVLNFPAEI

> Glyma20g34570.1 (GmERF8)

KSYRGVRRRPWGKFAAEIRDSTRHGMRVWLGTFDSAEAAALAYDQAAFSMRGSAAILNFPVEI

> Glyma10g33060.1 (GmERF9)

KSYRGVRRRPWGKFAAEIRDSTRHGMRVWLGTFDSAEAAALAYDQAAFSMRGSAAILNFPAEI

> Glyma17g15460.1 (GmERF10)

NHYRGVRQRPWGKFAAEIRDPNKRGSRVWLGTFDTAVEAAKAYDRAAFRLRGSKAILNFPLEV

>Glyma0041s00200.1

YRGVTRHHQHGRWQARIGRVAGNKDLYLGTFSTQEEAAEAYDVAAIKFRGVNAVTNFDIS

>Glyma01g02760.1

YRGVTRHHQQGRWQARIGRVAGNKDLYLGTFATEEEAAEAYDIAAIKFRGANAVTNFEMN

>Glyma01g03110.1

MRYRGVRRRPWGRYAAEIRDPQSKERRWLGTFDTAEEAACAYDCAARAMRGLKARTNFVYPT

>Glyma01g13410.1

PSYRGVRMRAWGKWVSEIREPRKKSRIWLGTYPTAEMAARAHDVAALAVKGHSAFLNFP

>Glyma01g20450.1

GKYKGVRMRKWGKWAAEIRDPFKGARIWLGTFSTAEEASQAYNARRLEFEAMAKALSDE

>Glyma01g24380.1

MCLSLLQRNTSPWGERRGRRKQAEPGKFLGLGTFDTAQEAALAYDRAALSMKGSQARTTFVY

>Glyma01g34280.1

PLHHDVRMRNWGKWVSEIREPWKKSRIWLGTFPTPEMAVWAHNVAALSIKGSAAILNFL

>Glyma01g35010.1

SVYRGVRMRTWGKWVSEIREPRKKNRIWLGTFATAEMAARAHDVAALTIKGSSAILNFP

>Glyma01g39520.1

YRGVTLHKCGRWEARMGQFLGKKYVYLGLFDTEIEAARAYDKAAIKCNGKEAVTNFDPSI

>Glyma01g39520.2

YRGVTLHKCGRWEARMGQFLGKKYVYLGLFDTEIEAARAYDKAAIKCNGKEAVTNFDPSI

>Glyma01g39540.1

RRYKGIRMRKWGKWVAEIREPNKRSRIWLGSYSTPVAAARAYDTAVFYLRGPSARLNFPELL

>Glyma01g40380.1

SQYRGVTRHRWTGRYEAHLWDNSCKKEGQGRKGRQVYLGGYDMEEKAARAYDMAALKYWGPSSHINF
PL

>Glyma01g42500.1

PVYRGVRRRNKNKWVCEMRVPNNNSRIWLGTYPTPEMAARAHDVAALALRGKSACLNFA

>Glyma01g42500.2

PVYRGVRRRNKNKWVCEMRVPNNNSRIWLGTYPTPEMAARAHDVAALALRGKSACLNFA

>Glyma01g42510.1

PLFHGIRCRGRKWVSEIREPRKASRIWLGTFPTPEMAAAAYDVAALALKGDGAVLNLPHSV

>Glyma01g43350.1

KKFRGVRQRPWGKWAAEIRDPSRRVRLWLGTYDTAEEAALVYDNAAIRLRGPHALTNFIT

>Glyma01g43450.1

KLYRGVRQRHWGKWVAEIRLPQNRMRVWLGTYDTAEAAAYAYDRAAYKLRGEYARLNFP

>Glyma01g44130.1

SSYRGVRQRKWGKWVSEIREPGKKSRIWLGSYESPEMAAAAYDVAALHLRGRAARLNFP

>Glyma01g44140.1

YRGVRKRKWGKWVSEIREPGTKTRIWLGSFETPEMAAAAYDVAALHFRGRDARLNFP

>Glyma01g44230.1

KKLKGVRRRKWGKWVSEIRVPGTQGRLWLGTYATQEAAAVAHDVAVYCLRRPSSLDKLNFPETL

>Glyma02g00870.1

SSFRGVRSRPWGKFAAEIRDSTRHGVRVWLGTFDSAEAAALAYDQAAFSMRGSAAVLNFPVE

>Glyma02g00890.1

VKYRGVRRRPWGKFAAEIRDSMRQGQRLWLGTFNTAEEAARAYDRAAYAMRGPFAVLNFP

>Glyma02g01960.1

NLYRGIRQRPWGKWAAEIRDPRKGVRVWLGTFNTAEEAARAYDREARKIRGKKAKVNFP

>Glyma02g04460.1

MRYRGVRRRPWGRYAAEIRDPQSKERRWLGTFDTAEEACAYDCAARAMRGLKARTNFVYP

>Glyma02g07310.1

KHYRGVRQRPWGKWAAEIRDPKKAARVWLGTFDTAEAAAMAYDAAALRFKGNKAKLNFPERV

>Glyma02g07460.1

GRYLGVRRRPWGRYAAEIRDPSTKERHWLGTFDTAEEAALAYDRAARSMRGSRARTNFVY

>Glyma02g08020.1

SKYVGVRQRASGKWVAEIKDTTQKIRMWLGTYETAEEAARAYDEAACLLRGSNTRTNFITRV

>Glyma02g09600.1

QYRGVTFYRRTGRWESHIWDCGKQVYLGGFDTAQAAARAYDRAAIKFRGVDADINFSL

>Glyma02g14940.1

KKYRGVRQRPSGKWAAEIRDRHRSARVWLGTFETAEDAARAYDKASFELRGPRAKLNFPL

>Glyma02g31350.1

CEYRGVRQRTWGKWVAEIREPKKRTRLWLGSFATAEEAALAYDEAARRLYGPDAYLNLP

>Glyma02g33090.1

SIYRGVTRHRWTGRYEAHLWDKSTWNQNQNKKGKQVYLGAYDDEEAAARAYDLAALRYWGPSALINFP
V

>Glyma02g36880.1

SIYRGVTRHRWTGRYEAHLWDKHCWNESQNKKGRQGAYDNEEAAAHAYDLAALKYWGQDTILNFPL

>Glyma02g40320.1

NKFRGVRQRPWGRWAAEIRDPTRRKRLWLGTFDTAEEAATEYDRAAVKLKGPNAVTNFPL

>Glyma02g42960.1

CNYRGVRQRTWGKWVGEIREPNRGSRLWLGTFSSAQEAALAYDEAARAMYGPCARLNFP

>Glyma02g43240.1

FRYRGVRQRSWGKWVAEIREPRKRTRKWLGTFATAEDAARAYDRAAIILYGSRAQLNLQPSG

>Glyma02g43500.1

TRFRGVRKRPWGRFAAEIRDPWKKQRVWLGTFDSAEDAARAYDKAARSFRGPKAKTNFPP

>Glyma02g46340.1

PRYRGVRKRPWGRFAAEIRDPLKKARVWLGTFDSAEDAARAYDAAARTLRGPKAKTNFP

>Glyma03g01930.1

FIGVRQRPSGRWVAEIKDSSQHVRLWLGTYDTPEEAARAYDEAARALR

>Glyma03g23330.1

HKFRGVRQRPWGRWAAEIRDPTRRTRVWLGTFDTAEEAAMVYDKAAIKFRGAEAVTNFI

>Glyma03g26330.1

VELPGPKEEAMGKVCSREISVRVGLGTYKTVENAALSYDRVAFKIHGSKTKLNFSHLI

>Glyma03g26390.1

KRFRGVRRRPWGKFAAEIWDPKKKNGRVWLGTYETEEEAGLAYDRACFKMRGSKAKLNFPHLI

>Glyma03g26450.1

KHYRGVRRRPWGKFAAEIWVPKSKGGRVWLGTYETEEEAGLAYDRAAFKMRGSKAKLNFPHLV

>Glyma03g26480.1

KHYRGVRRRPWGKFAAEIRDPNKNSARVWLGTYVTEEEAGLAYDRAAFKIHGSKAKLNFPHLI

>Glyma03g26510.1

TEIRDPKKNGARVWLGTYETEEEASLACDRAAFEMRGSKVKLNFPHLI

>Glyma03g26520.1

KRYRGVRRRPWGKFAAEIRDPKKNGARIWLGTYETEEEAGLAYDRAAFKMRGSKAKLNFPHLI

>Glyma03g26530.1

QNYKGVRRRPWGKFAAEIRDPNKNVRVWLGTYESAEDAALAYDRAAFEMRGSKAKLNFPHLI

>Glyma03g27050.1

PTYRGVRMRNWGKWVSEIREPRKKSRIWLGTYPTAEMAARAHDVAALAIKGHSAYLNFP

>Glyma03g29240.1

YRGVTRHRWTGRYEAHLWDKSTWNQNQNKKGKQGAYDDEEAAARAYDLAALKYWGPGTLINFPV

>Glyma03g29530.1

CEYRGVRQRTWGKWVAEIREPKKRTRLWLGSFATAEEAAMAYDEAARRLYGPDAYLNLP

>Glyma03g29680.1

YLGVYRNRTSENRKWIARLDREGLPTLHLGIFDTAEDAARAYDIISIKLNGWDALTNFHL

>Glyma03g31640.1

AHFRGVRKRPWGRFAAEIREPWKKTRKWLGTFDTAEEAARAYDAAARTLRGPKAKTNFSYIL

>Glyma03g31910.1

HGARVWLGTFNTAEEAARAYDRAAFEMRGTMAILNFPN

>Glyma03g31920.1

RTYRGVRSRPWGKFAAEIRDPTRNGVRVWIGTFVSAEEAALAYDQAAFLTRGVLATLNFSVQV

>Glyma03g31930.1

VRYRGIRRRPWGKFAAEIRDPTRKGARIWLGTFDTAEQAARAYDAAAFHFRGHKAILNFPNEY

>Glyma03g33470.1

YRGVTLHKCGRWEARMGQFLGKKYIYLGLFDSELEAARAYDKAAIKCNGREAVTNFEPS

>Glyma03g34970.1

PVYRGVRRRSSGKWVSEIREPKKPNRIWLGTFATPEMAAIAYDVAALALKGKDAELNFP

>Glyma03g41640.1

KRLRGVRQRPWGRWAAEIRDPVKRIRVWLGTYDTAEEAAMVYDKAAIAFRGSKALTNFI

>Glyma03g41910.1

PMYRGVRKRRWGKWVSEIREPRKKNRIWLGSFPVPEMAARAYDVAAYCLKGRKAQLNFP

>Glyma03g42450.1

NQYRGIRQRPWGKWAAEIRDPRKGVRVWLGTFNTAEEAARAYDAEARRIRGKKAKVNFP

>Glyma03g42450.2

NQYRGIRQRPWGKWAAEIRDPRKGVRVWLGTFNTAEEAARAYDAEARRIRGKKAKVNFP

>Glyma04g03070.1

CTYKGVRQRTWGKWVAEIREPNRGARLWLGTFETSHEAALAYDAAARKLYGSDAKLNLP

>Glyma04g05080.1

YRGVTRHHQHGRWQARIGRVAGNKDLYLGTFSTQEEAAEAYDVAAIKFRGANAVTNFDI

>Glyma04g06100.1

YKGVRKRKWGKWVSEIRLPNSRERIWLGSYDSPEKAARAFDAALYCLRGRHANFNFP

>Glyma04g06690.1

QRYRGVRQRHWGSWVSEIRHPILKTRIWLGTFETAEDAARAYDEAARLMCGTRARTNFPYNP

>Glyma04g07140.1

KKFRGVRQRQWGSWVSEIRHPLLKRRVWLGTFETAEAAARAYDQAAILMNGQNAKTNFP

>Glyma04g08900.1

PLYRGVRMRNWGKWVSEIREPRKKSRIWLGTFPTPEMAARAHDVAALSIKGPAAILNFP

>Glyma04g11210.1

PKFVGVRQRASGKWAADIKHTSKKIRLWLGTYQTAEEAARAYDEDACPLQGSNTATKGF

>Glyma04g11290.1

KLYRGVRQRHWGKWVAEIRLPKNRTRLWLGTFDTAEEAALAYDKAAYKLRGDFARLNFP

>Glyma04g19650.1

RKFRGVRQRHWGSWVSEIRHPLLKRRVWLGTFETAEEAARAYDQAAILMSGRNAKTNFPI

>Glyma04g21710.1

LKFCGIRQRPWGKWATEIWDLARRMCLWLGTYETAEEAIMVYDDTIIRLRGSDALTNFMLP

>Glyma04g24010.1

YRGVARHHHNGRWEARIGRVFGNKYLYLGTYATQEEAATAYDMAAIEYRGVNAVTNFDL

>Glyma04g37870.1

RHYRGVRQRPWGKWAAEIHDPKKAARVWLGTFETAEAAALAYDEAALRFKGSKAKLNFPERV

>Glyma04g37890.1

RHYRGVRQRPWGKWAAEIRDPKKAARVWLGTFETAEAAALAYDEAALRFKGSKAKLNFPERV

>Glyma04g39510.1

RKYRGVRQRPWGKWAAEIRDPFKAARVWLGTFETAEAAARAYDEAALRFRGSKAKLNFPENV

>Glyma04g41740.1

KKFRGVRQRPWGKWAAEIRDPARRVRLWLGTYDTAEEAAMVYDNAAIRLRGPDALTNFL

>Glyma05g03540.1

PLYHGIRCRGGKWVTEIREPRKTNRIWLGTFLTPEMAAAAYDVAALALKGGEAVLNFP

>Glyma05g03560.1

YRGVRRRNNNKWVCEVRVPNDKSTRIWLGTYPVPEMAARAHDVAALALRGKSACLNFA

>Glyma05g04920.1

PTYRGVRMRQWGKWVSEIREPRKKSRIWLGTFPTPDMAARAHDVAALTIKGSSAYLNFP

>Glyma05g07690.1

KKFLGVRQRPSGRWIAEIKDSSQKLRLWLGTFDKAEEAALAYDCAARLLRGRNAKTNFP

>Glyma05g18110.1

YRGVRWRNNNKWVCEVRVPNDKSTTIWLGTYPTPEMVTHAHDIATLALRGMSRLPLL

>Glyma05g18170.1

YRGVTLHKCGRWEARMGQFLGKKYVYLGLFDTEIEAARAYDKAAIKCNGKEAVTNFDPSI

>Glyma05g19050.1

TRYKGIRMRKWGKWVAEIREPNKRSRIWLGSYSTPVAAARAYDTAVFYLRGPSARLNFPELL

>Glyma05g22970.1

YRGVTSRHHQHGRWQARIGRVAGNKDLYLGTFSTQEEAAEAYDIAAIKFRGANAVTNFDI

>Glyma05g29010.1

PKFVGVRQRASGKWAAEIKDTSKKIRLWLGTYQTAEEAARAYDEAACLLRGSNTRTNFSTQG

>Glyma05g31370.1

KLYRGVRQRHWGKWVAEIRLPKNRTRLWLGTFDTAEEAALAYDNAAFKLRGEFARLNFP

>Glyma05g32040.1

RKYRGVRQRPWGKWAAEIRDPFKATRVWLGTFETAEDAARAYDQASLRFRGNKAKLNFPENV

>Glyma05g33440.1

CVGVRQRPWGKWASEIRDPKKAARVWLGTFETAEAAALAYDEAALRFKGTKAKLNFPERV

>Glyma05g35740.1

SVYRGVRMRAWGKWVSEIREPRKKNRIWLGTFATAEMAARAHDVAALAIKGNSAILNFP

>Glyma06g03110.1

CTYKGVRQRTWGKWVAEIREPNRGARLWLGTFETSHEAALAYDAAARKLYGSDAKLNLP

>Glyma06g04490.1

KPYRGIRMRKWGKWVAEIREPNKRSRIWLGSYATPVAAARAYDTAVFHLRGPSARLNFPELL

>Glyma06g05170.1

YRGVTRHHQHGRWQARIGRVAGNKDLYLGTFSTQEEAAEAYDIAAIKFRGANAVTNFDI

>Glyma06g06100.1

NLYKGVRKRKWGKWVSEIRLPNSRERIWLGSYDSPEKAARAFDAALYCLRGRHANFNFP

>Glyma06g06780.1

QRYRGVRQRHWGSWVSEIRHPILKTRIWLGTFETAEDAARAYDEAARLMCGTRARTNFPYNP

>Glyma06g07240.1

KKFRGVRQRQWGSWVSEIRHPLLKRRVWLGTFETAEAAARAYDQAAILMNGQNAKTNFP

>Glyma06g07240.2

KKFRGVRQRQWGSWVSEIRHPLLKRRVWLGTFETAEAAARAYDQAAILMNGQNAKTNFP

>Glyma06g08990.1

PLYRGVRMRNWGKWVSEIREPRKKSRIWLGTFPTPEMAARAHDVAALSIKGSAAILNFP

>Glyma06g11010.1

KLYRGVRQRHWGKWVAEIRLPKNRTRLWLGTFDTAEEAALAYDKAAYKLRGDFARLNFP

>Glyma06g11700.1

KKYKGVRMRSWGSWVSEIRAPNQKTRIWLGSYSTPEAAARAYDAALLCLKGSSANLNFPL

>Glyma06g13040.1

KKFRGVRQRPWGKWAAEIRDPARRVRLWLGTYDTAEEAAMVYDNAAIRLRGPDALTNFVTPP

>Glyma06g17180.1

RHYRGVRQRPWGKWAAEIRDPKKAARVWLGTFETAEAAALAYDEAALRFKGSKAKLNFPERV

>Glyma06g29110.1

RRVWLGTFETAEEAARAYDQAAILMSGRNAKTNFPI

>Glyma06g30840.1

YRGVARHHHNGRWEARIGRVFGNKYLYLGTYATQEEAATAYDMAAIEYRGLNAVTNFDLS

>Glyma06g37980.1

YRGVTRWTGRYEAHLWDNSCRRDRQTRKGRQGGYDKEEKAARAYDLAALKYRGTTTTTNFPV

>Glyma06g40010.1

HFRGVRKRSWGRYASEIRDPSKKSRVWLGTFDTAEEAARAYDAAAREFRDPKAKTNFPLPL

>Glyma06g44430.1

LHFRGVRKRPWGRYAAEIRDPGKKTRVWLGTFDTAEDAARAYDAAARNFRGPKAKTNFPVPP

>Glyma06g45010.1

KLYRGVRQRHWGKWVAEIRLPRNRTRLWLGTFDTAEDAAMAYDREAFKLRGENAKLNFP

>Glyma06g45680.1

CNYRGVRQRTWGKWVAEIREPNRGSRLWLGTFPTAISAALAYDEAARAMYGSCARLNFP

>Glyma07g02000.1

PVYHGVRKRNWGKWVSEIREPRKKSRIWLGTFSTPEMAARAHDVAALTIKGQSAILNFP

>Glyma07g02380.1

YRGVARHHHNGRWEARIGRVFGNKYLYLGTYATQEEAAAAYDMAAIEYRGLNAVTNFDL

>Glyma07g03040.1

KSYRGVRKRPWGRWSAEIRDRIGRCRHWLGTFDTAEEAARAYDAAARRMRGAKARTNFKI

>Glyma07g03500.1

RKFRGVRQRNWGSWVSEIRHPLLKRRVWLGTFETADEAARAYDEAAILMSGRNAKTNFPV

>Glyma07g04260.1

QYRGVTRHRWTGRYEAHLWDNSCRKEGQTRKGRQGGYDKEEKAAKAYDLAAIKYWGPTTHINFPL

>Glyma07g04950.1

NQYRGIRQRPWGKWAAEIRDPRKGVRVWLGTFSTAEEAARAYDAEARRIRGKKAKVNFP

>Glyma07g04950.2

NQYRGIRQRPWGKWAAEIRDPRKGVRVWLGTFSTAEEAARAYDAEARRIRGKKAKVNFP

>Glyma07g04950.3

NQYRGIRQRPWGKWAAEIRDPRKGVRVWLGTFSTAEEAARAYDAEARRIRGKKAKVNFP

>Glyma07g04950.4

NQYRGIRQRPWGKWAAEIRDPRKGVRVWLGTFSTAEEAARAYDAEARRIRGKKAKVNFP

>Glyma07g08540.1

RRFIGVRQRPSGRWVAEIKDSSQHVRLWLGTYDTPEEAARAYDEAARALR

>Glyma07g10120.1

SMYKGVQRRKWGKYVAEIKDPIRGVRMWLGTFDTEEEAVVAYERKRNEFDSSLLALSKRDAL

>Glyma07g13980.1

LSFRGVRRRPWGKYAAEIRDAKRNGVRVWLGTYETAENAALAYDRAAFKMHGSKAKLNFPHLI

>Glyma07g14060.1

KHYRGVRRRTWGKFAAEIRDPKKNGARIWLGTYETEEAAGLAYDRAAFKMRGSKAKLNFPHLI

>Glyma07g14070.1

QNYKGVRRRPWGKFAAEIRDPNRNGARVWLGTYNSAEDAALAYDRAAFEMRGSKAKLNFPHLI

>Glyma07g14560.1

PTYRGVRMRNWGKWVSEIREPRKKSRIWLGTYPTAEMAARAHDVAALAIKGHSAYLNFP

>Glyma07g19220.1

CKFRGVRQRIWGKWVAEIREPINGKLVGEKANRLWLGTFSTALEAALAYDEAAKAMYGPCARLNFPEPI

>Glyma07g31990.1

VHYRGVRKRRWGKDVAEIRNPNKKTRTWLGTFDSAIEAAKAWDVAA

>Glyma07g32000.1

MRKRWGKYGVYIRHPGKKILVWLGSFDSAIEAAKAYDAAAIKFCGFDKAKTNFSIP

>Glyma07g33510.1

KKYRGVRQRPWGKWAAEIRDPRRAARVWLGTFGTAEDAARAYDKAAIEFRGPRAKLNFPL

>Glyma07g37410.1

NVYRGIRQRPWGKWAAEIRDPRKGVRVWLGTFNTAEEAARAYDNAAKRIRGDKAKLNFP

>Glyma07g37990.1

GRFLGVRRRPWGRYAAEIRDPTTKERHWLGTFDTAQEAALAYDRAALSMKGSQARTNFV

>Glyma08g02460.1

KKFRGVRQRPWGKWAAEIRDPSRRVRLWLGTYDTAEEAAIVYDNAAIQLRGADALTNFI

>Glyma08g04550.1

SKYLGVRRRPWGKYAAEIRDPRQKNCRKRLWLGSYDTEIEAAMTFNVKRQEFEREMALERGDNASVHSE

>Glyma08g12130.1

PKFVGVRQRASGKWAAEIKDTSKKIRLWLGTYQTAEEAARAYDEAACLLRGSNTRTNFS

>Glyma08g14600.1

KLYRGVRQRHWGKWVAEIRLPKNRTRLWLGTFDTAEEAALAYDNAAFKLRGEFARLNFP

>Glyma08g15350.1

RKYRGVRQRPWGKWAAEIRDPLKARRVWLGTFETAEDAARAYDQASLRFRGNKAKLNFPENV

>Glyma08g15830.1

RKYRGVRQRKWGKWAAEIYNPFQSTRIWIGTFSTAEEASQAYEARRLEFEAMAKAQAYK

>Glyma08g21650.1

PVYHGVRKRNWGKWVSEIREPRKKSRIWLGTFATPEMAARAHDVAALTIKGESAILNFP

>Glyma08g22590.1

RKFRGVRQRHWGSWVSEIRHPLLKRRVWLGTFETAEEAARAYDEAAILMSGRNAKTNFPV

>Glyma08g23160.1

QRYRGVRQRHWGSWVSEIRHPLLKTRIWLGTFETAEDAARAYDEAARLMCGSKARTNFPYNP

>Glyma08g23630.1

YRGVARHHHNGRWEARIGRVFGNKYLYLGTYATQEEAAAAYDMAAIEYRGLNAVTNFDL

>Glyma08g23630.2

SIYRGVTRHRWTGRYEAHLWDKNCWNESQSKKGRQVYLGAYDDEEAAARAYDLAALKYWGQDTILNFP
L

>Glyma08g24420.1

IYRGVTRHRWTGRFEAHLWDKSSWNNIQSKKGKQGAYDTEESAARTYDLAALKYWGKDATLNFPI

>Glyma08g28820.1

PHYRGVRQRPWGKWAAEIRDPKKAARVWLGTFETAEDAALAYDKAALKFKGTKAKLNFPERL

>Glyma08g38170.1

VHFRGVRKRPWGRYASEIRDPSKKSRVWLGTFDTAEATTRAYDVAAREF

>Glyma08g38190.1

YRGVTRHHQQGRWQARIGRVAGNKDLYLGTFSTEEEAAEAYDIAAIKFRGSSAVTNFEM

>Glyma08g40830.1

TVKRSSRHRWTGRFEAHLWDKGTWNPTQKKKGKQGAYNDEEAAARAYDLAALKYWGISTFTNFPV

>Glyma08g43300.1

QRYRGVRKRPWGRFAAEIRDPLKKARVWLGTFDTAEEAARAYDTAARTLRGPKAKTNFPLS

>Glyma09g04630.1

NVYRGIRQRPWGKWAAEIRDPHKGVRVWLGTFPTAEEAAQAYDDAAIRIRGDKAKLNFPA

>Glyma09g05840.1

VKYRGVRRRPWGKFGAEIRDPTKPTGRQWLGTFDTAEEAARAYDRAAIELRGVLAILNFPDEC

>Glyma09g05850.1

VKYRGVRKRPWGKFGAEIRDPTKPTGRQWLGTFDTAEEAARAYDRAAIALRGALAILNFPHEF

>Glyma09g05860.1

VKYRGVRKRPWGKFGAEIRDPTKPTGRQWLGTFDTAEEAARAYDRAAIALRGALAILNFPHEF

>Glyma09g08330.1

SSYRGVRKRKWGKYVSEIRLPNSRQRIWLGSYDSAEKAARAFDAAMFCLRGSGAKFNFP

>Glyma09g27180.1

PVYRGVRRRNSDKWVCEVREPNKKTRIWLGTFPTPEMAARAHDVAAMALRGRYACLNFA

>Glyma09g32730.1

SVYRGVRMRTWGKWVSEIREPRKKNRIWLGTFATAEMAARAHDVAALTIKGSSAILNFP

>Glyma09g33240.1

YRGVTRHHQQGRWQARIGRVAGNKDLYLGTFATEEEAAEAYDIAAIKFRGANAVTNFEM

>Glyma09g36840.1

SAFRGVRKRSWGRYVSEIRLPGQKTRIWLGSFGSPEMAARAYDSAAFFLKGTSATLNFP

>Glyma09g37540.1

YRGVARHHHNGRWEARIGRVFGNKYLYLGTYSTQEEAARAYDIAAIEYRGIHAVTNFDL

>Glyma09g37780.1

KHYRGVRRRPWGKFAAEIRDPKKNGARVWLGTYDTEEKAALAYDKAAFKMRGRKAKLNFPHLI

>Glyma10g00980.1

SSFRGVRRRPWGKFAAEIRDSTRHGVRVWLGTFDNAEAAALAYDQAAFSMRGSGAVLNFPVE

>Glyma10g00990.1

VRYRGVRRRPWGKYAAEIRDPSKQGSRLWLGTFETGEEAARAYDHAAFTMRGHVAILNFPNEY

>Glyma10g02080.1

NLYRGIRQRPWGKWAAEIRDPRKGVRVWLGTFNTAEEAARAYDREARKIRGKKAKVNFP

>Glyma10g04160.1

PRYRGVRRRPWGKFAAEIRDPARHGARVWLGTFLTAEEAARAYDRAAYEMRGALAVLNFP

>Glyma10g04170.1

RSYIGVRKRPWGKFAAEIRDTTRNGTRVWLGTFDTAEAAALAYDQAAFSMRGQSAVLNFPVK

>Glyma10g04190.1

VRYRGIRRRPWGKFAAEIRDPTRKGTRIWLGTFDTAEQAARAYDAAAFHFRGHRAILNFPNEY

>Glyma10g04210.1

RPFRGVRRRPWGKFAAEIRDSTRNGVRVWIGTFDTAEAAALAYDQAALSTRGSMAVLNFPEEV

>Glyma10g07000.1

VHFRGVRKRPWGRYASKIRDPSQKSRVWLGTFDTAEATARAYDAAAREFRGPKAKTNFPLPL

>Glyma10g07740.1

YRGVRRRTSGKWVSEIREPKKPNRIWLGTFPTPEMAAVAYDVAALALKGKDAGLNFP

>Glyma10g10420.1

YAAEIGDSAHHGAKIWLGTFQTAEEAAMAYNIASFKMRGAKALLNFPSKL

>Glyma10g21850.1

CEYRGVRQRTWGKWVAEIREPKKRTRLWLGSFATAEEAALAYDEAARRLYGPDAYLNLP

>Glyma10g22390.1

SQYRGVTFYRRTGRWESHIWDCGKQVYLGGFDTAQAAARAYDRAAIKFRGVEADINFSL

>Glyma10g23440.1

KHYRGVRRRPWGKYAAEIRDPNRKGSRVWLGTFDTAIEAAKAYDKAAFKMRGSKAILNFPLEI

>Glyma10g24220.1

KHYRGVRQWPWGKFVVEIHDPNKCGSRVWLRTLDTTIKANKAYNQVIFRLHGSKANLNFPLEV

>Glyma10g31440.1

YRGVTRHHQHGRWQARIGRVAVNKDLYLGTFNTQEEAAEAYDIAAIKFRGLKAVTNFDM

>Glyma10g33070.1

VRFRGVRRRPWGKYAAEIRDPSKQGSRLWLGTFDTAEEAARAYDRAAFNLRGHLAILNFPS

>Glyma10g33080.1

VRYRGVRRRPWGKFAAEIRDSTRQGQRVWLGTFNTAEEAARAYDRAAYTMRGPFAILNFP

>Glyma10g33700.1

KLFKGVRQRHWGKWVAEIRLPRNRTRVWLGTFDTAEDAAIAYDTAAYILRGEYAQLNFP

>Glyma10g33810.1

RRYRGVRRRPWGKFAAEIRDPKKKGSRVWLGTFDTEIDAAKAYDCAAFRMRGHKAVLNFPLEA

>Glyma10g36300.1

TMYLGVRKRPWGRYAAEIRNPYTKERHWLGTFDTAEEAAIAYDLSSIKICGINARTNFHYPF

>Glyma10g36760.1

RRYRGVRQRPWGKWAAEIRDPHKAARVWLGTFDTAEAAARAYDEAALRFRGNRAKLNFPENV

>Glyma10g38420.1

FRYRGTRCRSGKWVSEIREPRKTNRIWLGTYPTAEMAAAAYDVAALALKGPDTPVNFP

>Glyma10g38440.1

PVYRGVRRRDSGKWVCEVREPNKKSRIWLGTFPTAEMAARAHDVAALALRGRSACLNFA

>Glyma10g42130.1

GKYRGVRQRKWGKWAAEIRDPFQCTRIWLGTFNTAEEASKAYETRRLEFEAMAKTQTL

>Glyma10g42130.2

GKYRGVRQRKWGKWAAEIRDPFQCTRIWLGTFNTAEEASKAYETRRLEFEAMAKTQTL

>Glyma11g01640.1

YRGVRKRKWGKWVSEIREPGTKTRIWLGSFETPEMAAAAYDVAALHFRGRDARLNFP

>Glyma11g02050.1

KLYRGVRQRHWGKWVAEIRLPQNRMRVWLGTYDTAEAAAYAYDRAAYKLRGEYARLNFP

>Glyma11g02140.1

KKFRGVRQRPWGKWAAEIRDPARRVRLWLGTYDTAEEAALVYDNAAIKLRGPHALTNFI

>Glyma11g03790.1

PTYRGVRMRKWGKWVSEIREPKKKSRIWLGSFSTPEMAARAHDVAALTIKGTSAFLNFP

>Glyma11g03910.1

KHYRGVRQRPWGKFAAEIRDPAKNGARVWLGTFETAEDAALAYDRAAYRMRGSRALLNFPLRI

>Glyma11g04910.1

YRGVTSRHHQHGRWQARIGRVAGNKDLYLGTFSTQEEAAEAYDIAAIKFRGVNAVTNFDIT

>Glyma11g05700.1

RRYKGIRMRKWGKWVAEIREPNKRSRIWLGSYSTPVAAARAYDTAVFYLRGPSARLNFPELL

>Glyma11g14040.1

YRGVTRHHQHGRWQARIGRVAGNKDLYLGTFSTEEEAAEAYDIAAIKFRGLNAVTNFDMS

>Glyma11g14040.2

YRGVTRHHQHGRWQARIGRVAGNKDLYLGTFSTEEEAAEAYDIAAIKFRGLNAVTNFDMS

>Glyma11g15650.1

YRGVTLHKCGRWEARMGQFLGKKYIYLGLFDSEVEAARAYDKAAIKCNGREAVTNFEPS

>Glyma11g15650.2

SQYRGVTFYRRTGRWESHIWDCGKQVYLGGFDTAHAAARAYDRAAIKFRGVDADINFNL

>Glyma11g15650.3

SQYRGVTFYRRTGRWESHIWDCGKQVYLGGFDTAHAAARAYDRAAIKFRGVDADINFNL

>Glyma11g18690.1

PRYWGMRKRLWGRFTVEIRDLLKKARVWLGTFDSAEDAARAYDIAAQTLRVEEE

>Glyma11g31400.1

NKFRGVRQRPWGRWTAEIRDPTQRKRVWLGTFDTAEEAAAVYDEAAVKLKGPNAVTNFPL

>Glyma12g06010.1

YRGVTRHHQHGRWQARIGRVAGNKDLYLGTFSTEEEAAEAYDIAAIKFRGLNAVTNFDM

>Glyma12g07800.1

SQYRGVTFYRRTGRWESHIWDCGKQVYLGGFDTAHAAARAYDRAAIKFRGVDADINFNL

>Glyma12g07800.2

SQYRGVTFYRRTGRWESHIWDCGKQVYLGGFDTAHAAARAYDRAAIKFRGVDADINFNL

>Glyma12g09130.1

PVYRGVRQRNRNKWVCEIREPIKKSRIWVGTYPTPEMAARAHDVAVLALSGTSANFNFP

>Glyma12g11150.1

CNYRGVRQRTWGKWVAEIREPNRGSRLWLGTFPTAISAALAYDEAAMAMYGFCARLNFP

>Glyma12g11150.2

CNYRGVRQRTWGKWVAEIREPNRGSRLWLGTFPTAISAALAYDEAAMAMYGFCARLNFP

>Glyma12g13320.1

VHFRGVRKRPWGRYAAEIRDPGKKTRVWLGTFDTAEDAARAYDVAARNFRGPKAKTTSP

>Glyma12g23070.1

YKGVVPQSNGHSGAQIYKKHQRVWVGTFNEEDKAARAYNIVAQHFRGRDAVTNLK

>Glyma12g26780.1

IRYRGVRKRPWGRYAAEIRDPGKKTRVWLGTFDTAEEAARAYDAAARDPSQSSTLDSSS

>Glyma12g30710.1

PIYRGVRQRKGKWVCELREPKKTTRIWLGTYPTPEMAARAHDVGALAIRGTSAILNFP

>Glyma12g30740.1

PVYRGVRQRNGNRWVCEVREPNKKSRIWLGTYPTPEMAARAHDVAVLALKGTSALFNFP

>Glyma12g32400.1

CNYRGVRQRTWGKWVAEIREPNRGNRLWLGTFPTAIGAALAYDEAARAMYGSCARLNFP

>Glyma12g33020.1

KLYRGVRQRHWGKWVAEIRLPRNRTRLWLGTFDTAEDAAMAYDREAFKLRGENARLNFP

>Glyma12g35550.1

IRYRGVRKRPWGRYAAEIRDPGKKTRVWLGTFDTAEEAARAYDTAAREFRGAKAKTNFP

>Glyma13g00950.1

YRGVTRHHQHGRWQARIGRVAGNKDLYLGTFSTQEEAAEAYDIAAIKFRGLNAVTNFDM

>Glyma13g01930.1

KLYRGVRQRHWGKWVAEIRLPKNRTRLWLGTFDTAEEAALAYDKAAYRLRGDLARLNFP

>Glyma13g02860.1

RSWGSWVSEIRAPNQKTRIWLGSYSTAEAAARAYDAALLCLKGSSATNLNFP

>Glyma13g05690.1

KRFVGVRQRPSGRWVAEIKDTIQKIRVWLGTFDTAEEAARAYDEAACLLRGTNTRTNFW

>Glyma13g08490.1

KKFRGVRQRPWGKWAAEIRDPVQRVRIWLGTFETAEEAALCYDNAAIMLRGPDALTNFGIRS

>Glyma13g17250.1

KYYKGVRKRKWGKWVSEIRLPNSRQRIWLGSFDTPEKAARAFDAAMFCLRGRNAKFNFP

>Glyma13g18330.1

PRYRGVRRRPWGKFAAEIRDPARHGARVWLGTFLTAEEAARAYDRAAYEMRGALAVLNFPNE

>Glyma13g18340.1

RSYIGVRKRPWGKFAAEIRDTTRNGTRVWLGTFESAEAAALAYDQAAFSMRGHDAVLNFPVK

>Glyma13g18350.1

RSYIGVRKRPWGKFAAEIRDTTRNGARVWLGTFDSAEAAALAYDQAAFTMRGDNAVLNFPVKT

>Glyma13g18370.1

RSYTGVRKRPWGKYAAEIRDTTRNGTRVWLGTFDTAEAAALAYDQAAFSMRGHNAVLNFPIKR

>Glyma13g18390.1

RSYIGVRKRPWGKFAAEIRDTTRNGARVWLGTFDSIEAAALAYDQAAFTMRGDHAVLNFPVKT

>Glyma13g18400.1

VRYRGIRRRPWGKFAAEIRDPTRKGTRIWLGTFDTAEQAARAYDAAAFHFRGHRAILNFP

>Glyma13g18410.1

RPFRGVRRRPWGKFAAEIRDSTRNGVRVWIGTFDTAEAAALAYDQAALSTRGSMAVLNFPEEV

>Glyma13g21560.1

YRGVRRRNSGKWVSEIREPKKPNRIWLGTFPTPEMAAVAYDVAALALKGKDAGLNFP

>Glyma13g23570.1

KKFRGVRQRHWGSWVSEIRHPLLKRRVWLGTFETAEEAARAYDQAAILMSGRNAKTNFPI

>Glyma13g28810.1

GRFLGVRRRPWGRYAAEIRNPLTKERHWLGTFDTAQEAALAYDRAALSMKGCQARTNFIY

>Glyma13g29920.1

HRFVGVRQRPSGRWVAEIKDSLQKVRLWLGTYDTAEDAARAYDNAARALRGSNARTNFEL

>Glyma13g30710.1

KHYRGVRRRPWGKYAAEIRDSSKKGARVWLGTFDTAEEAALAYDKAALRIRGPKAYLNFPLER

>Glyma13g30720.1

KHYRGVRRRPWGKYAAEIRDSARHGARIWLGTFQTAEEAAMAYDRAAFKMRGSKALLNFPAEI

>Glyma13g30990.1

VHFRGVRKRPWGRYAAEIRDPGKKSRVWLGTFDTAEEAARAYDAAAREFRGPKAKTNFPLPL

>Glyma13g31010.1

GHYRGVRKRPWGRYAAEIRDPWKKTRVWLGTFDTPEEAALAYDGAARSLRGAKAKTNFPPA

>Glyma13g34920.1

IRYRGVRKRPWGRYAAEIRDPGKKTRVWLGTFDTAEEAARAYDTAAREFRGAKAKTNFPT

>Glyma13g37450.1

KLYRGVRQRHWGKWVAEIRLPRNRTRLWLGTFDTAEDAAMAYDREAFKQRGENARLNFPELF

>Glyma13g38030.1

CNYRGVRQRTWGKWVAEIREPNRGNRLWLGTFPTAIGAALAYDEAARAMYGSCARLNFP

>Glyma13g39540.1

PVYRGVRQRNGNKWVCEVREPNKKSRIWLGTYPSPEMAARAHDVAVLALKGTSAVFNFP

>Glyma13g40470.1

YRGVTLHKCGRWEARMGQFLGKKYIYLGLFDSEVEAARAYDKAAIKCNGREAVTNFEPS

>Glyma13g40470.3

IYLGLFDSEVEAARAYDKAAIKCNGREAVTNF

>Glyma13g44660.1

QRYRGVRQRHWGSWVSEIRHPLLKTRIWLGTFETAEDAARAYDEAARLMCGPKARTNFPY

>Glyma14g02360.1

PRYRGVRKRPWGRFAAEIRDPLKKARVWLGTFDSAEDAARAYDTAARNLRGSKAKTNFPL

>Glyma14g05470.1

TRFRGVRKRPWGRFAAEIRDPWKKQRVWLGTFDSAEDAARAYDKAARSFRGPKAKTNFP

>Glyma14g05470.2

TRFRGVRKRPWGRFAAEIRDPWKKQRVWLGTFDSAEDAARAYDKAARSFRGPKAKTNFP

>Glyma14g06080.1

CNYRGVRQRTWGKWVGEIREPNRGSRLWLGTFSSAQEAALAYDEAARAMYGPCARLNFP

>Glyma14g06290.1

FRYRGVRQRSWGKWVAEIREPRKRTRKWLGTFATAEDAARAYDRAAIILYGSRAQLNLQPSG

>Glyma14g07620.1

CTYKGVRQRTWGKWVAEIREPNRGARLWLGTFDTAREAALAYDAAARKLYGPDAKLNLA

>Glyma14g09320.1

KPYRGIRMRKWGKWVAEIREPNKRSRIWLGSYTTPVAAARAYDTAVFYLRGPTARLNFPELL

>Glyma14g10130.1

YRGVTRHHQHGRWQARIGRVAGNKDLYLGTFSTQEEAAEAYDIAAIKFRGLNAVTNFDI

>Glyma14g13470.1

QRYRGVRQRHWGSWVSEIRHPILKTRIWLGTFETAEDAARAYDEAARLMCGARARTNFPF

>Glyma14g13890.1

KLYRRVRQRHWGKWVTEISLPKNRTRLWLGTFDTIEEAALVYDNTAFKLRGKFARLNFP

>Glyma14g22740.1

PVYRGVRMRNWGKWVSEIREPRKKSRIWLGTFPTPEMAARAHDVAALSIKGNSAILNFP

>Glyma14g22970.1

KKYKGVRMRSWGSWVSEIRAPNQKTRIWLGSYSTAEAAARAYDAALLCLKGSSANLNFPSSS

>Glyma14g27060.1

HFRGVRKRPWGRYASEIRDPSKKSRVWLGTFDTAEEAARAYDGAT

>Glyma14g29040.1

RKFRGVRQRPWGKWAAEIRDPVQRVRIWLGTFKTAEEAALCYDNAAITLRGPDALTNFG

>Glyma14g34590.1

KLYRGVRQRHWGKWVAEIRLPKNRTRLWLGTFDTAEEAALAYDKAAYRLRGDFARLNFPSLK

>Glyma14g38610.1

NKFRGVRQRQWGRWAAEIRDPTRRKRLWLGTFDTAEEAATEYDRAAVKLKGPNAVTNFPL

>Glyma15g00660.1

QRYRGVRQRHWGSWVSEIRHPLLKTRIWLGTFETAEDAARAYDEAARLMCGPKARTNFPYNP

>Glyma15g01140.1

KKFRGVRQRHWGSWVSEIRHPLLKRRVWLGTFGTAEEAARAYDDAAILMSGRNAKTNFPVA

>Glyma15g02130.1

PDYHGVRMRNWGKWVSEIREPRKKSRIWLGTFATPEMAARAHDVAALSIKGHTAILNFP

>Glyma15g02900.1

PVYRGVRRRSSGKWVSEIREPKKPNRIWLGTFATPEMAAIAYDVAALALKGKDAELNFP

>Glyma15g04930.1

YRGVTLHKCGRWEARMGQFLGKKYIYLGLFDSEVEAARAYDKAAIKCNRREAVTNFEPSI

>Glyma15g04930.2

YRGVTLHKCGRWEARMGQFLGKKYIYLGLFDSEVEAARAYDKAAIKCNRREAVTNFEPSI

>Glyma15g08360.1

GHYRGVRKRPWGRYAAEIRDPWKKTRVWLGTFDTPEEAALAYDGAARSLRGAKAKTNFPPA

>Glyma15g08370.1

LHFRGVRKRPWGRFAAEIRDPAKKTRVWLGTFDTAEEAARAYDAAAREFRGPKAKTNFPLPS

>Glyma15g08560.1

KHYRGVRRRPWGKYAAEIRDSARHGARIWLGTFQTAEEAAMAYDRAAFKMRGSKALLNFPAEI

>Glyma15g08580.1

KHYRGVRRRPWGKYAAEIRDSSKKGARVWLGTFDTAEEAALSYDKAALRIRGPKAYLNFPLEM

>Glyma15g09190.1

HRFVGVRQRPSGRWVAEIKDSLQKVRLWLGTFDTAEDAARAYDNAARALRGANARTNFEL

>Glyma15g10250.1

GRFLGVRRRPWGRYAAEIRNPLTKERHWLGTFDTAQEAALAYDRAALSMKGCQARTNFIYSK

>Glyma15g16260.1

NVYRGIRQRPWGKWAAEIRDPHKGVRVWLGTFPTAEEAARAYDDAAKRIRGDKAKLNFP

>Glyma15g17100.1

VKYRGVRRRPWGKFGAEIRDPTKPTGRQWLGTFDTAEEAARAYDRAAIGLRGALAILNFPDEY

>Glyma15g19910.1

IVHVGVRKRKWGKYVSEIRLPNSRQRIWLGSYDSAEKAARAFDAAMFCLRGSGANFNFP

>Glyma15g23560.1

KTRIWLGTFETVEGATRAYDEAVRLMCGTRARTNFPY

>Glyma15g34770.1

SIYRGVTRHRWTGRFEAHLWDKSSWNNIQSKKGRQGAYDTEESAARTYDLAALKYWGKDATLNFPI

>Glyma16g00950.1

YRGVTRHHQHGRWQARIGRVAGNKDLYLGTFSTQEEAAEAYDIAAIKFRGTSAVTNFDI

>Glyma16g01500.1

NQYRGIRQRPWGKWAAEIRDPRKGVRVWLGTFSTAEEAARAYDAEARRIRGKKAKVNFP

>Glyma16g01500.2

NQYRGIRQRPWGKWAAEIRDPRKGVRVWLGTFSTAEEAARAYDAEARRIRGKKAKVNFP

>Glyma16g01500.3

NQYRGIRQRPWGKWAAEIRDPRKGVRVWLGTFSTAEEAARAYDAEARRIRGKKAKVNFP

>Glyma16g01500.4

NQYRGIRQRPWGKWAAEIRDPRKGVRVWLGTFSTAEEAARAYDAEARRIRGKKAKVNFP

>Glyma16g02680.1

PLFRGVRKRRWGKWVSEIREPRKKSRIWLGSFPAPEMAAKAYDVAAYCLKGRKAQLNFPDEV

>Glyma16g04410.1

NKFVGVRQRPSGRWVAEIKDTTQKIRMWLGTFETAEEAARAYDEAACLLRGSNTRTNFITHV

>Glyma16g05070.1

GRYLGVRRRPWGRYAAEIRDPSTKERHWLGTFDTADEAALAYDRAARAMRGSRARTNFVYA

>Glyma16g05190.1

RHYRGVRQRPWGKWAAEIRDPKKAARVWLGTFDTAEAAAAAYDAAALKFKGSKAKLNFPEHV

>Glyma16g08690.1

HKFRGVRQRPWGRWAAEIRDPLRRTRVWLGTFDTAEEAAMVYDKAAIKFRGAEAVTNFI

>Glyma16g26460.1

GRYLGVRRRPWGRYAAEIRDPSTKERHWLGTFDTAEEAALAYDKAARSMRGSRARTNFIY

>Glyma16g27040.1

SKYVGVRQRASGKWVAEIKDTTQKIRMWLGTYETAEEAARAYDEAACLLRGSNTRTNFITRV

>Glyma16g27950.1

RKYRGVRQRPWGKWAAEIRDPHKAARVWLGTFDTEEAAARAYDEAALRFRGNRAKLNFP

>Glyma16g32330.1

VYRGVRRRNTDKWVSEVREPNKKTRIWLGTFPTPEMAARAHDVAAMALRGRYACLNFA

>Glyma17g02710.1

GRFLGVRRRPWGRYAAEIRDPTTKERHWLGTFDTAQEAALAYDRAALSMKGSQARTNFV

>Glyma17g05240.1

KYYKGVRKRKWGKWVSEIRLPNSRQRIWLGSYDTPEKAARAFDAAMFCLRGRNAKFNFP

>Glyma17g07010.1

YRGVTRHHQHGRWQARIGRVAGNKDLYLGTFSTQEEAAEAYDIAAIKFRGLNAVTNFDM

>Glyma17g12330.1

KKFRGVRQRHWGSWVSEIRHPLLKRRVWLGTFETAEEAARAYDQAAILMSGRNAKTNFPI

>Glyma17g13320.1

KKFLGVRQRPSGRWIAEIKDSSQKLRLWLGTFDKAEEAALAYDCAARLLRGRNAKTNFP

>Glyma17g14100.1

PLYHGIRCRGGKWVTEIREPRKTNRIWLGTFLTPEMAAAAYDVAALALKGNEAVLNFP

>Glyma17g14110.1

PVYRGVRRRNNNKWVCEVRVPNDKSTRIWLGTYPTPEMAARAHDVAALSLRGKSACLNFA

>Glyma17g15310.1

PTYRGVRMRQWGKWVSEIREPRKKSRIWLGTFPTPDMAARAHDVAALTIKGSSAYLNFP

>Glyma17g15480.1

KHYRGVRQRPWGKFAAEIRDPAKNGARVWLGTFETAEDAALAYDRAAYRMRGSRALLNFPLRV

>Glyma17g16080.1

PKFVGVRQRASGKWAADIKDTSKKIRLWLGTYQTVEEAARAYDEDVCLLRGSNTRTNFFTQ

>Glyma17g18580.1

TRYKGIRMRKWGKWVAEIREPNKRSRIWLGSYSTPVAAARAYDTAVFHLRGPSARLNFPELV

>Glyma17g18610.1

GKYRGVRQRKWGKWASEIYNPFQNNRIWLGTFNTAEEASQVYEARRLELEAMAKAQAYKT

>Glyma17g18640.1

SQYRGVTFYRRTGRWESHIWDCGKQVYLGGFDTAHAAARAYDRAAIKFRGVEADINFNI

>Glyma17g18640.2

SQYRGVTFYRRTGRWESHIWDCGKQVYLGGFDTAHAAARAYDRAAIKFRGVEADINFNI

>Glyma17g27520.1

PVYRGVRMRNWGKWVSEIREPRKKSRIWLGTFPTPEMAARAHDVAALGIKGNNAILNFP

>Glyma17g31900.1

KKFRGVRQRQWGSWVSEIRHPLLKRRVWLGTFETAEAAARAYDQAAILMSGQNAKTNFPTQM

>Glyma17g33060.1

QRYRGVRQRHWGSWVSEIRHPILKTRIWLGTFETAEDAARAYDEAARLMCGARARTNFPFNP

>Glyma17g33530.1

FKGVRKRKWGKWVSEIRLPNSRERIWLGSYDTQVKAARAFDAALYCLRGQSATFNFP

>Glyma17g35860.1

KPYRGIRMRKWGKWVAEIREPNKRSRIWLGSYTTPMAAARAYDTAVFYLRGPTARLNFP

>Glyma17g37350.1

CTYKGVRQRTWGKWVAEIREPNRGARLWLGTFDTAREAALAYDAAARKLYGPDAKLNLP

>Glyma18g02170.1

KLYRGVRQRHWGKWVAEIRLPKNRTRLWLGTFDTAEEAALAYDNAAFKLRGENARLNFP

>Glyma18g10290.1

PRYRGVRKRPWGRFAAEIRDPLKKARVWLGTFDTAEEAARAYDTAARTLRGPKAKTNFPLS

>Glyma18g16240.1

YRGVARHHHNGRWEARIGRVFGNKYLYLGTYSTQEEAARAYDIAAIEYRGINAVTNFDL

>Glyma18g20960.1

RYRGVRRRPWGRYAAEIRDPQSKERRWLGTFDTAEEAACAYDYAARAMRGAKARTNFVY

>Glyma18g43750.1

CKFRGVRQRIWGKWVAEIREPINGKLVGEKANRLWLGTFSTALEAALAYDEAAKALYGPCARLNFSESI

>Glyma18g47980.1

YRGVTRHHQHGRWQARIGRVAGNKDLYLGTFSTQEEAAEAYDVAAIKFRGLSAVTNFDM

>Glyma18g48720.1

TRYKGVRRRAHGKFAAEITDPNKNGRVWLGTYDTEEEAALAYDNAAFKIRGSKSKLNFPHLI

>Glyma18g48730.1

KHYRGVRRRPWGKFAAEIRDPKKNGARVWLGTYDTEEKAALAYDKAAFKMRGQKAKLNFPHLI

>Glyma18g48740.1

RRYRGVRRRPWGKFAAEIRDPKKNGSRVWLGTYVNEEEAALAYDKAAFNMRGQKAKLNFPHLI

>Glyma18g49760.1

KKFVGVRQRPSGRWVAEIKDTIQKIRVWLGTFDTAEEAARAYDEAACLLRGANTRTNFW

>Glyma18g51680.1

PHYRGVRQRPWGKWAAEIRDPKKAARVWLGTFETAEDAALAYDKAALKFKGTKAKLNFPERV

>Glyma19g03120.1

KRFVGVRQRPSGRWVAEIKDTIQKIRVWLGTFDTAEEAARAYDEAACLLRGANTRTNFW

>Glyma19g03170.1

KRFVGVRQRPSGRWVAEIKDTIQKIRVWLGTFDTAEEAARAYDEAACLLHGANMRTNFW

>Glyma19g04420.1

CEYRGVRQQTWGKWLGSFATAEEAAMAYDEAARRLYGPDAYLNLPHLQ

>Glyma19g27790.1

RHYRGVRQRPWGKWAAEIRDPKKAARVWLGTFDTAEAAAAAYDAAALKFKGSKAKLNFPEHV

>Glyma19g29000.1

NKFVGVRQRPSGRWVAEIKDTTQKIRMWLGTFETAEEAARAYDEAACLLRGSNTRTNFITHV

>Glyma19g32380.1

CEYRGVRQRTWGKWVAEIREPKKRTRLWLGSFATAEEAAMAYDEAARRLYGPDAYLNLP

>Glyma19g34650.1

IKYRGVRRRPWGKFAAEIRDSARHGARVWLGTFNTAEEAARAYDRAAFEMRGATAILNFPD

>Glyma19g34660.1

RRRPWGKFVAEKRDPTRNGVRVWTGAFDTAEAAALAYDQAAFLTRGVLATLNFSVQV

>Glyma19g34670.1

KSYIGVRRRPWGRFAAEIRDTTRKGIRVWLGTFDSAEEAALAYDQAAFSMRGSSAVLNFPVK

>Glyma19g34680.1

GKLQQRSVTPTIKGTRIWLDFDTAEQAARAYDTAAFHFRGHKAILN

>Glyma19g34690.1

KMYRGVRKRPWGKFAAEIRDSTRNGVRVWIGTFDTAEAAALAYDQAAFSTRGSLAVLNFPEEV

>Glyma19g36200.1

YRGVTLHKCGRWEARMGQFLGKKYIYLGLFDSELEAARAYDKAAIKCNGREAVTNFEPSL

>Glyma19g37670.1

PVYRGVRRRSSGKWVSEIREPKKPNRIWLGTFATPEMAAIAYDVAALALKGKDAELNFP

>Glyma19g40070.1

NLYRGIRQRPWGKWAAEIRDPRKGVRVWLGTFNTAEEAARAYDKEARKIRGKKAKVNFP

>Glyma19g44240.1

KKLRGIRQRPWGRWAAEIRDPVKRRRVWLGTYDTAEEAAMVYDKAAITFRGSNARTNFI

>Glyma19g44580.1

PVYRGVRKRRWGKWVSEIREPRKKNRIWLGSFPVPEMAARAYDVAAYCLKGRKAHLNFPDEV

>Glyma19g45200.1

NQYRGIRQRPWGKWAAEIRDPRKGVRVWLGTFNTAEEAARAYDAEARRIRGKKAKVNFPEEA

>Glyma20g03890.1

KRYIGVRQRPSGRWVSEIKDTIQNIRLWLGTYDTAEDAARAYDEAARLLRGANTRTNFF

>Glyma20g24920.1

GKFRGVRQRKWGKWAAEIRDPFQSTRIWLGTFNTAEEASQAYEARRLEFEAMAEAQAYK

>Glyma20g24920.2

GKFRGVRQRKWGKWAAEIRDPFQSTRIWLGTFNTAEEASQAYEARRLEFEAMAEAQAYK

>Glyma20g29410.1

PVYRGVRRRDSGKWVCEVREPNKKSRIWLGTFPTAEMAARAHDVAAIALRGRSACLNFA

>Glyma20g29440.1

VRYRGTRYRSGKWVSEIREPRKTKRIWLGTYPTAEMAAAAYDVAALALKGPDTPLNFP

>Glyma20g30840.1

IRDPHKAARVWLGTFDTAEAAARAYDEAALRFRGNRAKLNFPENV

>Glyma20g31300.1

YLGVRKRPWGRYAAEIRNPYTKERRWLGTFDTAEEAAIAYDLSSIKICGINARTNFHYP

>Glyma20g33800.1

RHYRGVRRRPWGKFAAEIRDPKKKGSRVWLGTFDTEIDAAKAYDCAAFRMRGHKAVLNFPLEA

>Glyma20g33840.1

TCYRGVRQRPWGKFTAEIRDPARNGARAWLGTYQTAEDAALAYDRAAFKLRGSKALLNFPHRI

>Glyma20g33890.1

KLFKGVRQRHWGKWVAEIRLPRNRTRVWLGTFDSAEDAAIAYDTAAYILRGEYAQLNFP

>Glyma20g34550.1

VRYRGVRRRPWGKFAAEIRDSTRQGQRVWLGTFNTAEEAARAYDRAAYAMRGPFAILNFPDEY

>Glyma20g34560.1

VRYRGVRRRPWGKYAAEIRDPSKQGSRLWLGTFDTAEEAARAYDRAAFNLRGHLAILNFPS

>Glyma20g35820.1

QKFRGVRQRPLGKWSAEIRDPSQRGVRLWLGTYNTAEEAALVYDNAAIKLRGPHALTNFI

>ERF I

LYRGVRQRHWGKWVAEIRLPRNRTRLWLGTFDTAEEAALAYDKAAYKLRGDFARLNFP

>ERF II

RYKGIRMRKWGKWVAEIREPNKRSRIWLGSYKTAVAAARAYDTAVFYLRGPSARLNFP

>ERF III

IYRGVRQRNSGKWVSEVREPNKKTRIWLGTFQTAEMAARAHDVAALALRGRSACLNFA

>ERF IV

SFRGVRQRIWGKWVAEIREPNRGSRLWLGTFPTAQEAASAYDEAAKAMYGPLARLNFP

>ERF V

KFRGVRQRHWGSWVAEIRHPLLKRRIWLGTFETAEEAARAYDEAAVLMSGRNAKTNFP

>ERF VI

KFRGVRQRPWGKWAAEIRDPSRRVRVWLGTFDTAEEAAIVYDNAAIQLRGPNAELNFP

>ERF VII

VYRGIRKRPWGKWAAEIRDPRKGVRVWLGTFNTAEEAAMAYDVAAKQIRGDKAKLNFP

>ERF VIII

RFLGVRRRPWGRYAAEIRDPTTKERHWLGTFDTAEEAALAYDRAARSMRGTRARTNFV

>ERF IX

HYRGVRQRPWGKFAAEIRDPAKNGARVWLGTFETAEDAALAYDRAAFRMRGSRALLNFP

>ERF X

KYRGVRQRPWGKWAAEIRDPHKATRVWLGTFETAEAAARAYDAAALRFRGSKAKLNFP

>ERF VI-L

KPVGVRQRKWGKWAAEIRHPITKVRTWLGTYETLEQAADAYATKKLAFDALAAATSAA

>ERF XB-L

KHKGVRKKPSGKWAAEIWDPSLKVRRWLGTFPTAEMAAKAYNDAAAEFVGRRSARRGT

>AP2 first

SSVHRGVTRHRWTGRYEAHLWDKNSWNETQTKKGRQVYLGAYDEEDAAARAYDLAALKYWGRDTILN
FP

>AP2 first

TSIYRGVTRHRWTGRYEAHLWDNSCRREGQSRKGRQVYLGGYDKEEKAARAYDLAALKYRGLNAVTNF
E

>AP2 second

VSKYRGVAKHHHNGRWEARIGRVFGNKYLYLGTYATQEEAAIAYDIAAIEYRGLNAVTNFDISRYL

>AP2 second

ASIYRGVTRHHQHGRWQARIGRVAGNKDLYLGTFGTQEEAAEAYDVAAIKFRGTNAVTNFDITRYD

>AP2 first

SSQYRGVTFYRRTGRWESHIWDCGKQVYLGGFDTAHAAARAYDRAAIKFRGVDADINFDIEDYL
