# Supplementary material for: High level transgenic expression of soybean (Glycine max) GmERF and Gmubi gene promoters isolated by a novel promoter analysis pipeline
Source: BMC Plant Biol. 2010 Nov 4;10:237. doi: 10.1186/1471-2229-10-237 (PMC3095320; doi:10.1186/1471-2229-10-237)

Additional file 4. Regression analysis of GFP expression and transgene copy number scored on the Southern blots. GFP expression for each hairy root shown on Figure 7 was quantified and grayscale values correlated with the respective transgene copy number. The regression analysis showed P-Values of 0.005 and 0.039 for GmERF6- and GmERF10-containing hairy roots, respectively.

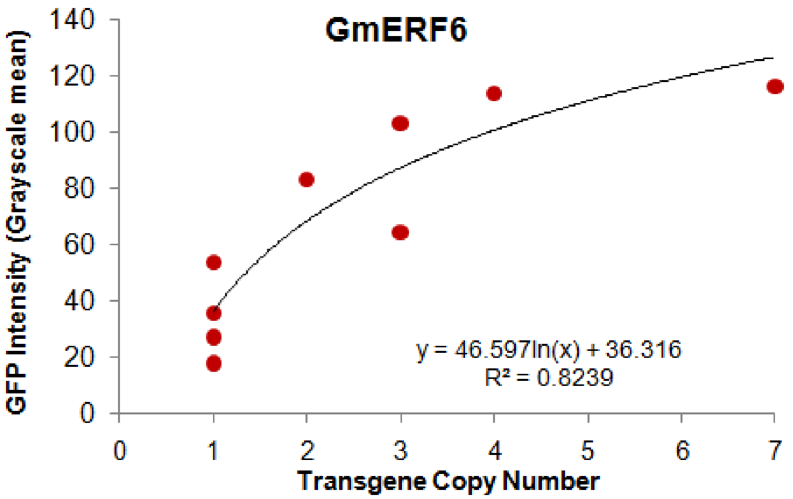

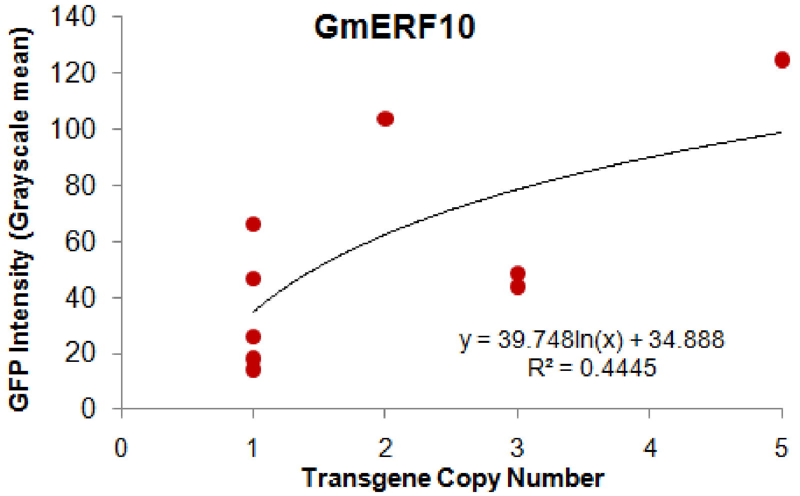

Supplement: Additional file 4 — Regression analysis of GFP expression and transgene copy number scored on the Southern blots. GFP expression for each hairy root shown on Figure 7 was quantified and grayscale values correlated with the respective transgene copy number. The regression analysis showed P-Values of 0.005 and 0.039 for GmERF6- and GmERF10-containing hairy roots, respectively. [file 1471-2229-10-237-S4.PDF]
