# Supplementary material for: High level transgenic expression of soybean (Glycine max) GmERF and Gmubi gene promoters isolated by a novel promoter analysis pipeline
Source: BMC Plant Biol. 2010 Nov 4;10:237. doi: 10.1186/1471-2229-10-237 (PMC3095320; doi:10.1186/1471-2229-10-237)
Supplement: Additional file 5 — List of primer sequences used to PCR-amplify the soybean promoters. Restriction sites incorporated in the forward and reverse primers are underlined. F forward primer, R reverse primer. [file 1471-2229-10-237-S5.PDF]

Additional file 5. List of primer sequences used to PCR-amplify the soybean promoters.

Restriction sites incorporated in the forward and reverse primers are underlined. *F* forward primer, *R* reverse primer.

Promoter	Primer sequence (5'→3')
Gmubi1	F: CACCCTGCAGAACAATTAGATGATACCCATTGCCCTTACG
	R: CACCCCATGGTACCTGTAGAGTCAACAATCACAAGATAAATCA
Gmubi2	F: CACCAAGCTTGGAAGGAGGCGCAGGATGGAGTACCAGTC
	R: CACCGCATGCTGGTACCTGTTCAATCACAAAAAAGAAACATC
Gmubi3	F: CACCCTGCAGTCCTTAAGTTGCAGCATTTAACACATCTCCTC
	R: CACCCCATGGTACCTGTCGAGTCAACAATCACAGATAAATCAGAA
Gmubi4	F: CACCAAGCTTCAAGCAGAACACTGAGACATAGAGG
	R: CACCCCATGGTACCTACATATACAATACAGTCAACAAAATAAATGAA
Gmubi5	F: CACCAAGCTTGGTTGTGACTTGATGCATTGTAAC
	R: CACCCCATGGTACCTACACATACAATACAGTCAACAAAAT
Gmubi6	F: CACCAAGCTTAGAAGTGTATTTCATACACTACTTTC
	R: CACCCCATGGTACCTATAAAACAAGATACGATAAAATTAATCACC
Gmubi7	F: CACCAAGCTTCATACCATTGAGATCCTTGTTGAGTG
	R: CACCGCATGCTGGTACCTGTTCAATCACAAACAGAACCAT
Gmubi8	F: CACCCTGCAGTCCCTAATTAATGTGATATTGGGAAAGATACG
	R: ATCTCCATGGCGAGATTGGGTATAATAAGCTTC
Gmubi9	F: CACCCTGCAGTCCATTATTCGGGATTTAGCAAATTG
	R: ATCTCCATGGTGAAAACGAGAATAATAGAGAGATATTTTAGTGGTGTAGG
Gmubi10	F: CACCAAGCTTGCCCCGCGAAACTTAAAAATGTT
	R: CACCCCATGGTGGCTAGCTAGGGTTTCTCTCAG
GmERF1	F: CACCAAGCTTATCTATTTGATGTGTAACAACACC
	R: GTTGCCATGGTTAAGGGATCTGAGTGATGAAAC
GmERF2	F: CACCAAGCTTGAACCTAAATCCGTAGCTGTTG
	R: CTTTCCATGGTTGGTTGTGAAATTGAGGCTTATAAGG
GmERF3	F: CACCAAGCTTTAATCTCATGCACGCGAC
	R: TTTGCCATGGGATATTGAGAGGAGAAGGAGCTTTAG

GmERF4	F: CACCAAGCTTCATAGACCATTTCTCCATCCTTTATC
	R: TTTGCCATGGGATATTGAGGGGAGAAGAGAGCG
GmERF5	F: CACCAAGCTTCGGACCAAACGGAGAAG
	R: CCGTCCATGGCTTCGGTGTTCCTTTTGTGGTC
GmERF6	F: CACCAAGCTTATGAGCCATCCCCGTTC
	R: TTAGCCATGGCTTAAGTTAGAAGCGCTTTTAGCTTG
GmERF7	F: CACCAAGCTTGACCCAGTATACAGTGCATGCACAAAG
	R: GAACCCATGGTCAACTCCTAATTTGAGTGTATGCAAAG
GmERF8[a]	F: CACCAAGCTTATAACCCGACAAATATAACATGTATAGATTAGC
	R: GAATCCATGGGTTAGAGTGTGTAAGGTAAGGGGAAACAG
GmERF9	F: CACCAAGCTTATAACCCGACAAATATAACATGTATAGATTAGC
	R: GAATCCATGGGTTAGAGTGTGTAAGGTAAGGGGAAACAG
GmERF10	F: CACCGCATGCATCATAGAACCCACCTTAATAATCTC
	R: TTCGCCATGGCTTAAGTTAGAAAGCGCTTTTCGCTTC

[a]GmERF8 was amplified using the same primers for GmERF9 amplification and it was further identified as a group IX ERF gene promoter.
